# Material Extrusion‐Enabled Soft Multimodal Pressure Sensors With Fabrication‐Embedded Stretch‐Induced Strain Insensitivity

**DOI:** 10.1002/smll.74747

**Published:** 2026-07-21

**Authors:** Jinsheng Fan, Shujia Xu, Yi Yang, David Harmon, Yuelin Deng, Brittany Newell, Wenzhuo Wu, Robert A. Nawrocki

**Affiliations:** ^1^ School of Engineering Technology Purdue University West Lafayette Indiana USA; ^2^ School of Industrial Engineering Purdue University West Lafayette Indiana USA; ^3^ College of Engineering Purdue University West Lafayette Indiana USA

**Keywords:** additive manufacturing, electrospinning, multimodal pressure sensing, piezoelectric polymer, soft grippers, stretchable electronics

## Abstract

Key challenges in stretchable pressure sensors include establishing cost‐effective fabrication routes for soft functional materials, achieving strain‐insensitive performance under deformation, and enabling seamless integration into soft architectures. Here, electrospinning is combined with liquid‐based material extrusion (*l*‐MEX) and melt‐based material extrusion (*m*‐MEX) to fabricate stretchable pressure sensors with multimodal sensing capability. The sensing layer is composed of mechanically anisotropic electrospun poly(vinylidene fluoride) / thermoplastic polyurethane composite microfibers sandwiched between *l*‐MEX printed stretchable silver electrodes, exhibiting a Young's modulus as low as 0.5 MPa. A three‐dimensional serpentine architecture reconfigures the sensing layer into a compliant geometry and enables system‐level stretchability while maintaining stable sensing performance under strain. In the capacitive mode, the optimized sensor achieves 97.6% strain insensitivity under strains up to 30% and a minimum detectable pressure of 0.04 kPa. In the piezoelectric mode, the same device exhibits a sensitivity of 16.5 mV kPa^−1^ and a strain‐insensitivity ratio of 85.4% at 10% applied strain. A stretchable capacitive sensing matrix enables uniform pressure mapping under strain. Integration of the sensors into a pneumatically actuated soft gripper further demonstrates real‐time piezoelectric responses. This work presents a scalable and automatable manufacturing route for integrating soft functional materials into stretchable electronics and soft robotic systems.

## Graphical abstract

Material extrusion‐based additive manufacturing integrates mechanically anisotropic electrospun piezoelectric composite microfibers with stretchable silver electrodes into a three‐dimensional serpentine architecture to realize stretch‐induced strain‐insensitive pressure sensors, maintaining a stable sensing performance under tensile strains up to 30%. This work bridges the gap between structural flexibility, electronic functionality, and system‐level integration, establishing a scalable pathway toward fully additively manufactured functional materials for soft robotic systems.

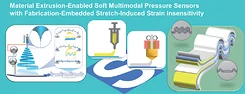

## 1 Introduction

Soft robotics has emerged as a prominent field in modern scientific and technological advancements [1, 2]. The ability to manipulate fragile objects with a human‐like delicate touch makes soft robotic systems highly desirable for a wide range of applications, enabled, in part, by integrated pressure sensing [3, 4]. The development of flexible and stretchable pressure sensors is both critically important and technically challenging. Since soft robotics exhibits expansive degrees of freedom and high mechanical compliance, integrated pressure sensors must be lightweight, omnidirectionally stretchable, and capable of conforming to or integrating within soft architectures [5, 6, 7]. A fundamental challenge in stretchable pressure sensors is to design and build mechanically compliant sensor arrays capable of enduring significant deformation while maintaining stable electrical functionality [8, 9]. In particular, longitudinal strain often induces compression in the sensing layer, producing deformation similar to applied pressure and leading to signal interference arising from deformations in both the electrodes and the sensing materials.

Recent studies have reported the development of stretchable pressure sensors based on piezoelectric, piezoresistive, and capacitive sensing mechanisms [10, 11, 12]. Piezoresistive sensors transduce applied pressure into resistance variations arising from changes in contact area or conductive networks within the sensing material. These devices typically employ deformable conductive layers composed of polymer matrices embedded with conductive nanofillers such as carbon nanotubes (CNTs) [13], graphene [14], or intrinsically conductive elastomers [15], which are sandwiched between flexible electrodes. However, planar elastomer composites face persistent challenges in achieving uniform filler dispersion and optimized interfacial structures. As a result, simultaneously achieving high sensitivity, a wide detection range, and fast response times remains difficult due to intrinsic material and structural constraints. Engineered microstructures have been introduced to address these limitations by enhancing deformability, redistributing stress, and increasing the effective contact area, thereby amplifying resistance variation and improving sensing performance across a broad pressure range [13, 16]. Nevertheless, microstructured piezoresistive designs commonly rely on mold‐based fabrication, which increases process complexity and cost [17].

To enhance sensitivity, the incorporation of microstructures into capacitive pressure sensors was first proposed in 2010 [18]. Capacitive sensors function by detecting variations in capacitance that arise from the deformation of the dielectric layer or the change in distance between electrodes under applied pressure. In 2021, a representative study reported a strain‐unperturbed capacitive sensor with superior strain stability by combining an electrical double‐layer‐based interfacial detection system with a pyramid microstructure featuring a stiffness hierarchy. The device exhibited 98% strain insensitivity up to 50% strain and a detection limit as low as 0.2 Pa [8]. Capacitive sensors possess excellent sensitivity, linearity, and temperature invariance. Despite these advantages, capacitive sensors typically require complex readout circuitry, which complicates device integration and miniaturization. Moreover, conductive elastomers used in capacitive devices remain susceptible to signal interference under in‐plane stretching, and electromagnetic interference often necessitates additional shielding to maintain a high signal‐to‐noise ratio [18, 19].

Piezoelectric pressure sensors directly convert mechanical deformation into electrical charge, enabling self‐powered sensing without external power sources [20, 21]. Applied pressure changes dipole alignment and spacing within the piezoelectric material, producing variations in internal electric fields that generate output charge and voltage [22]. These characteristics provide high sensitivity and rapid response to dynamic pressures, making piezoelectric sensors particularly attractive for miniaturized systems. A stretchable piezoelectric sensor employing an island–bridge architecture with lead zirconate titanate elements and serpentine electrodes achieved a detection threshold of 0.005 Pa [23]. However, rigid piezoelectric ceramics are difficult to process into complex device architectures. Compared with inorganic piezoelectric ceramics, piezoelectric polymers offer physical flexibility, superior processability, and mechanical compliance, enabling simpler device architectures and lower cost fabrication [22].

Fabrication challenges persist across all sensing mechanisms. Piezoresistive and capacitive sensors often rely on micro‐electro‐mechanical systems (MEMS)‐based mold fabrication to create microstructures critical for sensing performance [24, 25]. In piezoelectric devices, achieving high sensitivity (∼kPa^−1^) requires electrical poling to align dipoles normal to the film plane [26]. At the same time, device flexibility is frequently constrained by the intrinsic mechanical properties of functional materials, complicating seamless integration with soft robotic systems.

Additive manufacturing has emerged as a powerful technique for fabricating functional materials and devices, including flexible sensors [27, 28, 29, 30], electronic components [31, 32, 33], biomedical systems [34, 35], and artificial tissues [36, 37]. By enabling mold‐free fabrication of complex architectures, additive manufacturing reduces fabrication time and process complexity while simultaneously expanding design freedom. For example, Lee et al. employed high‐resolution additively manufactured multiscale substrates combined with gas‐phase conformal polymer coating to create a mold‐free piezoresistive sensor with a sensitivity of 184.8 kPa^−1^ [38]. Peng et al. reported a fully additively manufactured stretchable piezoresistive sensor with a hierarchically porous sensing element, achieving high sensitivity (5.5 kPa^−1^), a wide pressure range (10 Pa–800 kPa), and only 7% resistance variation under 50% strain [39]. Furthermore, pioneering studies by Nawrocki et al. demonstrated the combination of liquid‐based material extrusion (*l*‐MEX) and melt‐based material extrusion (*m*‐MEX) as an all‐additive manufacturing technique to fabricate fully additively manufactured piezoelectric pressure sensors [40, 41] and capacitive temperature sensors [42]. Building on this concept, Newell et al. employed multimaterial additive manufacturing techniques to fabricate pressure sensors directly embedded within pneumatic actuators [43]. Collectively, the combination of multiple MEX techniques establishes a promising pathway for the development of soft robotic systems in which fabrication‐embedded, soft functional materials are seamlessly integrated with three‐dimensional (3D) architectures. Such systems leverage the advantages of soft functional materials to achieve tailored electromechanical performance, drawing inspiration from biological systems.

Achieving high performance (∼kPa^−1^ sensitivity) in piezoelectric devices requires electrical poling to maximize polarization normal to the film plane. Contact poling and corona poling are the primary post‐processing techniques used to induce polarization after printing. Building on additive manufacturing platforms, researchers have developed an electric poling‐assisted additive manufacturing technique that enables direct printing of flexible, piezoelectrically active poly(vinylidene fluoride) (PVdF) materials [44, 45]. However, the piezoelectric performance of printed PVdF remains limited, and rigid thin‐film configurations often restrict mechanical compliance. In 2025, the application of *l*‐MEX to print flexible electrodes onto electrospun PVdF microfibers was proposed as an all‐additive manufacturing technique, demonstrating the combination of electrospinning and *l*‐MEX to realize fully additively manufactured flexible piezoelectric pressure sensors [46]. Electrospinning, an electric field‐assisted fiber fabrication technique, is compatible with other MEX processes and can be implemented within a unified manufacturing platform. Electrospinning enables the direct fabrication of piezoelectric polymer microfibers with high flexibility, biocompatibility, and cost efficiency [47]. Nevertheless, most electrospun piezoelectric pressure sensors still adopt planar thin‐film geometries, in which overall stretchability remains limited by the intrinsic mechanical properties and elongation limits of the constituent materials.

A key strategy for achieving high stretchability in electronic systems is the engineering of structural form factors of existing functional materials, devices, and fabrication methods [48, 49]. While most inorganic materials fracture at strains of approximately 1%, bio‐integrated electronic systems must accommodate strains comparable to those of biological tissues, which can typically reach around 20% [50]. This mismatch between intrinsic material properties and application requirements has motivated extensive efforts to impart stretchability through structural design rather than through material replacement. Since 2005, pre‐strained substrates combined with flexible thin films have been widely adopted to generate buckled or serpentine geometries upon strain release, enabling reversible deformation on both planar and curvilinear surfaces [51, 52, 53]. Among various design strategies, serpentine architectures are particularly effective for constructing continuous and stretchable structures from intrinsically stiff materials by patterning them into serpentine ribbon geometries on polymer substrates [54]. When the substrate is stretched, the serpentine ribbons accommodate the stretch‐induced strain through a combination of in‐plane rotation and out‐of‐plane buckling. This deformation mode reduces the effective stiffness, maintains low local strain within the active sensing materials, and prevents mechanical failure. Representative examples include ultrathin silicon or gold films patterned on pre‐strained elastomers, which achieve stretchability far beyond the intrinsic fracture strain of the constituent materials [55]. Additional approaches include stretchable conductive composites [56] and thin conductive films deposited on elastomeric substrates [57]. Beyond substrate‐supported systems, freestanding serpentine architectures have also been explored in deployable sensor arrays [58] and biomedical stents [59]. These systems offer increased geometric freedom and mechanical compliance. However, their fabrication typically relies on two‐dimensional planar processes such as spin coating, photolithography, and metal deposition [60]. These methods require expensive cleanroom facilities and impose inherent limitations on scalability, geometric complexity, and material integration.

Integrating electrospun piezoelectric fibers with additively manufactured freestanding serpentine architectures provides a promising pathway to address these limitations by combining material‐level functionality with geometry‐driven stretchability. The flexible and stretchable sensor architecture presented in this study is inspired by freestanding serpentine networks and the widely adopted island‐bridge design [61]. In this approach, planar sensing membranes are reconfigured into stretchable serpentine geometries through integration with soft 3D architectures. This configuration enables monolithic deformation along prescribed directions without inducing mechanical damage to the sensing layer. The geometry of the supporting 3D architecture governs the overall mechanical compliance and strain distribution of the system, providing a robust design strategy for optimizing stretchability and sensing performance.

Unlike many reported flexible pressure sensors that rely on molding, lithography, and coating processes, this work integrates electrospun piezoelectric microfibers, *l*‐MEX printed stretchable electrodes, and *m*‐MEX printed soft architectures within a unified additive manufacturing platform. More importantly, stretchable piezoelectric pressure sensing remains challenging because the piezoelectric output is strongly affected by local stress redistribution, microfiber deformation, electrode‐sensing‐layer contact, and structural strain transfer. By embedding the piezoelectric sensing layer into a strain‐accommodating 3D architecture, this approach expands the manufacturing strategy for soft robotic systems that require integrated dynamic pressure‐sensing capability.

In this study, electrospinning was combined with MEX‐based additive manufacturing techniques to fabricate stretchable pressure sensors with fabrication‐embedded, stretch‐induced strain insensitivity. The electrospun sensing layer consists of poly(vinylidene fluoride) (PVdF)/thermoplastic polyurethane (TPU) composite microfibers sandwiched between *l*‐MEX printed silver electrodes. Mechanical compliance was achieved through three complementary strategies: (1) incorporation of TPU to reduce the Young's modulus of PVdF from 16.0 MPa at 0 wt% TPU to 7.8 MPa at 50 wt% TPU, accompanied by a decrease in piezoelectric output from 2.6 to 0.5 pC N^−1^; (2) gap electrospinning to produce mechanically anisotropic PVdF/TPU microfibers with a modulus as low as 0.5 MPa measured perpendicular to the fiber axis; and (3) printing of stretchable silver‐paste electrodes. Integration with an *m*‐MEX printed serpentine TPU architecture reconfigured the sensing layer into a stretchable serpentine geometry, enabling the local strain to remain within the elastic limit of the sensing material. Pressure sensing performance was evaluated under applied strains of up to 30%. A stretchable capacitive sensing matrix achieved uniform spatial pressure mapping under strain, while a proof‐of‐concept pneumatically driven soft gripper integrated with the stretchable sensors exhibited real‐time piezoelectric voltage responses. By combining electrospinning, *l*‐MEX, and *m*‐MEX within a unified manufacturing framework, this work establishes a scalable and automatable platform for stretchable electronic systems in soft robotics and related applications.

## 2 Strategies for Improving Flexibility in Electrospun PVdF/TPU Composite Microfiber Sensing Layers

The core sensing layer of the flexible and stretchable pressure sensor consisted of electrospun piezoelectric PVdF‐based composite microfibers sandwiched between two *l*‐MEX printed silver electrodes. To reduce the Young's modulus of the sensing layer and enhance mechanical flexibility, three strategies were implemented: (1) incorporation of soft TPU, which exhibits a lower Young's modulus than PVdF, into the electrospinning solution to produce PVdF/TPU composite microfibers; (2) application of gap electrospinning to generate well‐aligned microfibers with anisotropic mechanical properties; and (3) electrode patterning using a stretchable conductive silver paste printed via *l*‐MEX.

**FIGURE 1:**
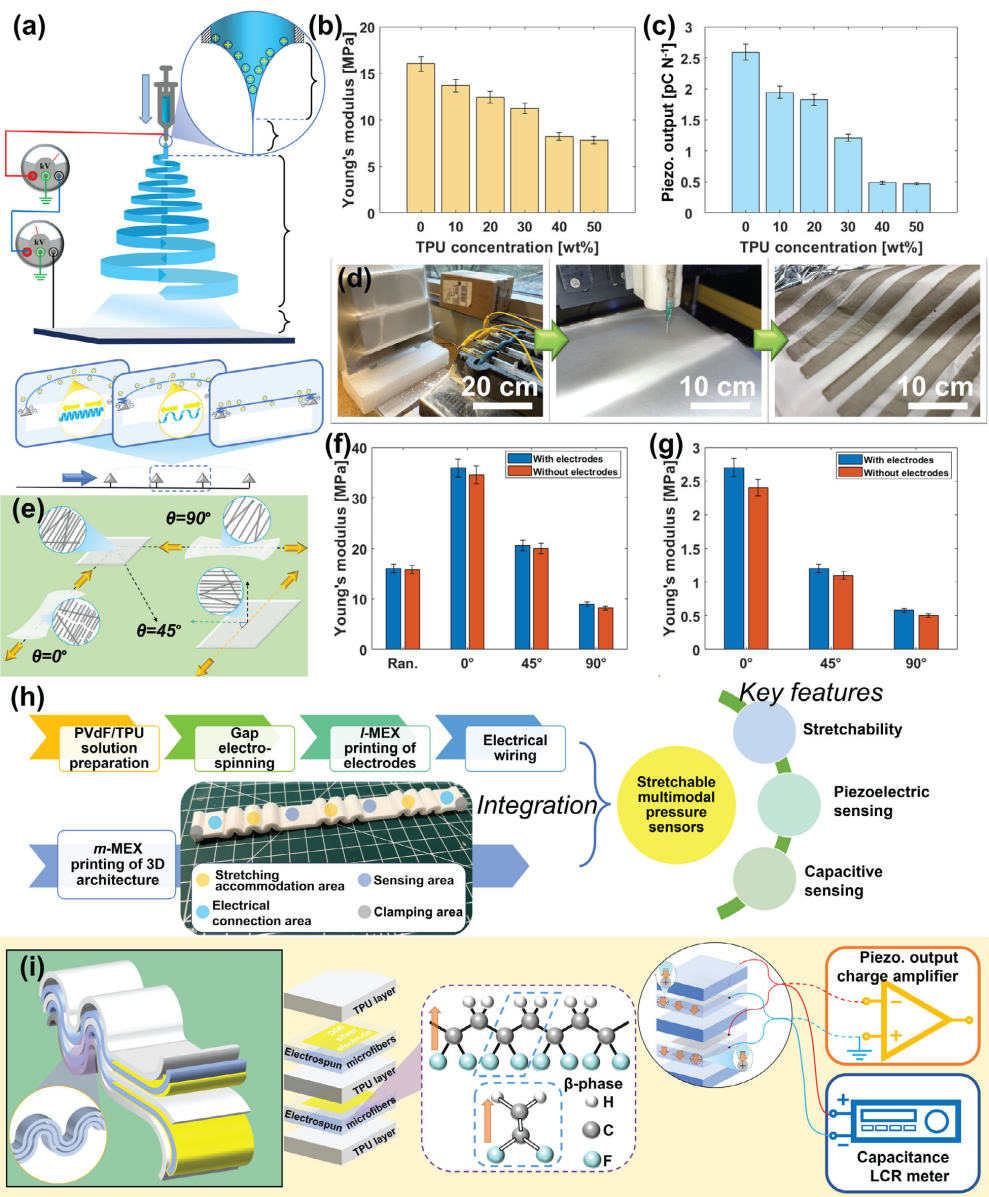
(a) Schematic illustration of the gap electrospinning setup and the mechanism for producing aligned microfibers. (b) Young's modulus and (c) piezoelectric output of electrospun PVdF/TPU microfibers with varying TPU concentrations. (d) Fabrication procedures for generating well‐aligned microfibers via gap electrospinning and subsequent *l*‐MEX printing of stretchable silver electrodes on the surface. (e) Definition of fiber alignment and the evolution of aligned fibers under different tensile loading conditions. Young's modulus of (f) PVdF microfibers and (g) PVdF/TPU microfibers with random and aligned orientations, measured with and without *l*‐MEX printed silver electrodes. (h) Flowchart summarizing the complete additive manufacturing process for fabricating the soft, stretchable pressure sensor, including a 3D architecture printed by *m*‐MEX that illustrates the functional segmentation of different regions within the device. (i) Layer‐by‐layer structural schematic of the PVdF/TPU microfiber‐based multimodal pressure sensor, including the two spatially stacked sensing layers, their parallel electrical connection, the *β*‐phase PVdF chain conformation and dipole orientation, and simplified signal‐acquisition schematics showing capacitance measurement using an inductance–capacitance–resistance meter (LCR meter) and piezoelectric signal acquisition using a charge amplifier.

A schematic of a conventional electrospinning setup is shown in Figure 1a. PVdF solutions containing various TPU concentrations were prepared to produce electrospun PVdF/TPU composite microfibers with random orientations. Flexibility was quantified by measuring Young's modulus via tensile testing, where a lower modulus corresponds to reduced resistance to deformation under an applied tensile load. As shown in Figure 1b, pure electrospun PVdF microfibers exhibited a Young's modulus of 16.0 MPa. Increasing the TPU concentration from 0 wt% to 30 wt% reduced the modulus to 11.2 MPa, while further increases to 40 wt% and 50 wt% TPU decreased the modulus to 8.2 MPa and 7.8 MPa, respectively. At TPU concentrations of 40 wt% or higher, the modulus was nearly halved relative to that of pure electrospun PVdF microfibers. TPU incorporation did not require adjustment of the electrospinning parameters optimized for pure PVdF, indicating good material compatibility during the electrospinning process. Compared with bulk PVdF films fabricated by *m*‐MEX (∼10 GPa) [44, 62], electrospun PVdF microfibers (∼10 MPa) exhibited an approximately three‐order‐of‐magnitude reduction in modulus due to the fibrous microstructure. These results indicated that modulating TPU concentration provided an effective strategy for tuning microfiber stiffness for soft robotic applications.

The upper limit of the TPU concentration was constrained by the piezoelectric output of electrospun PVdF/TPU composite microfibers. As shown in Figure 1c, piezoelectric output decreased monotonically with increasing TPU concentration. Pure PVdF microfibers generated approximately 2.6 pC N^−1^, while the addition of 10 wt% and 20 wt% TPU reduced the output to 1.9 pC N^−1^ and 1.8 pC N^−1^, respectively. At 30 wt% and 40 wt% TPU, the output decreased to 1.2 pC N^−1^ and 0.5 pC N^−1^ and remained at 0.5 pC N^−1^ at 50 wt% TPU. TPU concentrations above 50 wt% resulted in complete suppression of the measurable piezoelectric output. The combined results in Figure 1b,c demonstrated a trade‐off between reduced Young's modulus and piezoelectric output, indicating the need for additional structural or processing strategies to further lower stiffness without sacrificing electromechanical response.

From a microstructural perspective, random fiber orientations produced by conventional electrospinning led to extensive fiber entanglement, which limited further reductions in the modulus. To overcome this limitation, gap electrospinning was employed to produce well‐aligned electrospun microfibers exhibiting anisotropic mechanical behavior [63]. A schematic illustration of the fiber alignment mechanism during gap electrospinning is shown in Figure 1a. Unlike conventional electrospinning techniques that relied on high‐speed rotating collectors, where fiber alignment typically required rotational speeds exceeding 4000 rpm (corresponding to surface velocities greater than 416 m s^−1^) [64], gap electrospinning achieved fiber alignment through controlled electric fields, enabling the production of continuous fibers ranging from several centimeters to over one meter in length [65]. Representative fabrication steps combining gap electrospinning with *l*‐MEX printed silver electrodes are illustrated in Figure 1d.

To evaluate anisotropic mechanical behavior, tensile tests were conducted with tensile loading applied at 0°, 45°, and 90° relative to the microfiber length axis, as shown in Figure 1e. The results are shown in Figure 1f. Randomly oriented PVdF microfibers exhibited a Young's modulus of 16.0 MPa. In contrast, aligned electrospun PVdF microfibers displayed pronounced anisotropy. When tensile loading was applied parallel to the fiber length axis (0°), the modulus reached 34.6 MPa, approximately twice that of randomly oriented fibers. At loading angles of 45° and 90°, the modulus decreased to 20.0 MPa and 8.2 MPa, respectively. These results demonstrated that tensile loading parallel to the fiber length axis produced the highest resistance to deformation, whereas tensile loading perpendicular to the fiber length axis yielded the lowest modulus. The minimum modulus observed at 90° demonstrated the effectiveness of gap electrospinning for producing flexible, piezoelectrically active PVdF microfibers with reduced stiffness relative to randomly oriented counterparts.

The successful production of aligned electrospun PVdF microfibers with the fiber length axis parallel to the collector gap‐width direction established a critical guideline for subsequent device architecture design. To maximize flexibility and minimize resistance to applied strain, sensor architectures were therefore designed such that external stretching was applied perpendicular to the fiber length axis of aligned microfibers, thereby minimizing mechanical constraints imposed on the core sensing layer. Based on these findings, a PVdF solution containing 50 wt% TPU was prepared to fabricate aligned PVdF/TPU composite microfibers. As shown in Figure 1g, the Young's moduli of aligned PVdF/TPU microfibers tested at 0°, 45°, and 90° were 2.4 MPa, 1.1 MPa, and 0.5 MPa, respectively. These results demonstrated that the combined incorporation of TPU and gap electrospinning enabled the fabrication of piezoelectrically active microfibers with a Young's modulus as low as 0.5 MPa, while maintaining pronounced piezoelectric output (0.5 pC N^−1^).

The third strategy focused on preserving low stiffness after the electrode patterning of the sensing layer using a stretchable conductive silver paste printed via *l*‐MEX. The *l*‐MEX process enabled precise two‐dimensional electrode patterning and surface metallization required for sensor fabrication. The variation in electrical resistance of *l*‐MEX printed stretchable electrodes as a function of applied strain was investigated and described in detail in Section S1. Figure 1f compares the Young's moduli of aligned pure PVdF microfibers, with and without *l*‐MEX printed electrodes, to those of randomly oriented PVdF microfibers. Similarly, Figure 1g presents the moduli of aligned PVdF/TPU composite microfibers (50 wt% TPU), with and without electrodes, measured along three representative directions. Printing silver electrodes increased the Young's modulus of the core sensing layer by approximately 1.5%–16.0%, depending on microfiber orientation and electrode infill direction. Despite this increase, the core sensing layer retained sufficient mechanical compliance for flexible device fabrication.

It should be noted that, from a practical perspective, electrospinning provides an effective route for directly producing flexible, piezoelectrically active materials. However, electrospun sensing layers fabricated in planar geometries inherently limited sensor design freedom and complicated integration with soft robotic systems. In such planar configurations, overall stretchability was limited by the intrinsic mechanical properties and elongation limits of the constituent materials. System‐level stretchability was achieved by integrating the electrospun sensing layer with a stretchable 3D architecture printed by *m*‐MEX, as described in detail in the following section.

## 3 Combined Functional Material Fabrication Techniques for Stretch‐Induced Strain‐Insensitive Pressure Sensors

The key innovation of this study lay in the development of an all‐additive manufacturing technique that integrated piezoelectric functionality, mechanical compliance, and electrical interconnection within a single device, thereby enabling stretch‐induced strain‐insensitive pressure sensing that could not be achieved by any individual fabrication technique alone. Inspired by earlier strategies in which ultrathin silicon or gold films patterned on pre‐strained substrates formed buckled geometries upon strain release, enabling stretchability in two‐ and 3D configurations [55, 61], electrospun PVdF/TPU composite microfibers were reconfigured into buckled 3D architectures that accommodated large stretch‐induced strain without exceeding the elastic limit, thereby imparting system‐level stretchability.

A flowchart of the fabrication processes combining gap electrospinning, *l*‐MEX, and *m*‐MEX is shown in Figure 1h. The electrospun core sensing layer provided intrinsic flexibility, defined by a Young's modulus below 10^3^ MPa, while system‐level stretchability of the sensing device arose from the integration of the electrospun sensing layer with the soft TPU architecture, which reconfigured the sensing layer into a 3D serpentine geometry. Reconfiguration of the sensing layer into a buckled geometry enabled the device to accommodate large macroscopic strains while maintaining the local strain in the constituent materials below their yield limits. The detailed fabrication processes consisted of four sequential steps: (1) gap electrospinning to produce aligned, piezoelectrically active PVdF/TPU composite microfibers; (2) *l*‐MEX printing of stretchable silver‐paste electrodes onto the microfiber membrane to form the core sensing layer; (3) *m*‐MEX printing of a soft TPU 3D architecture; and (4) integration of the core sensing layer with the soft TPU 3D architecture and electrical wiring to complete device fabrication.

The stretchability of the *m*‐MEX printed 3D architecture arose from the use of intrinsically soft TPU filaments (Shore hardness 60 A) printed in an engineered serpentine architecture. The selected soft TPU filament represented one of the most compliant materials available for *m*‐MEX and was widely employed in applications requiring high flexibility and extensibility, including soft robotic actuators and components. However, the extreme softness of the filament introduced challenges during MEX‐based printing, including buckling and feeding instabilities. To address these challenges, a modified *m*‐MEX 3D printer was employed to enable reliable printing of the soft TPU filament.

The fabricated flexible and stretchable pressure sensor comprised four functional regions: (1) pressure sensing, (2) strain accommodation, (3) electrical interconnection, and (4) mechanical clamping. The device exhibited multimodal sensing capability, operating under both piezoelectric and capacitive sensing modes. The piezoelectric sensing mode, enabled by the electrospun piezoelectric PVdF‐based core sensing layer shown in Figure 1i, was designed for dynamic pressure detection. Under dynamic mechanical loading, deformation of the electrospun PVdF/TPU microfiber layer changed the polarization state of the piezoelectric dipoles, generating charge at the electrodes. The generated charge was converted into a measurable voltage using a charge amplifier circuit. Dynamic pressure was defined as pressure applied at frequencies higher than the low‐cutoff frequency of the signal‐conditioning circuit, as described in Figure S3. To enhance piezoelectric output, two core sensing layers were electrically connected in parallel and spatially stacked, yielding a higher signal amplitude than either a single layer or two layers connected in series. In this parallel configuration, the piezoelectric charges generated by the two sensing layers contributed to the measured output. The design of the capacitive mode was based on the structure of the core sensing layer consisting of a dielectric layer sandwiched between two electrodes, and the two sensing layers electrically connected in parallel formed parallel‐connected capacitors. Applied pressure compressed the dielectric structure and changed the effective electrode spacing and contact configuration, resulting in a capacitance change that was measured using an inductance–capacitance–resistance (LCR) meter. The corresponding signal‐acquisition pathways for the capacitive and piezoelectric modes are schematically illustrated in Figure 1i, and the detailed charge‐amplifier circuit is provided in Figure S3. The stretchable pressure sensor operated in a capacitive sensing mode for static pressure detection. Static pressure corresponded to pressure applied at frequencies below the low‐cutoff frequency of the signal‐conditioning circuit. Because the capacitance change was maintained under sustained deformation, the capacitive mode was suitable for static and slowly varying pressure measurements, whereas the piezoelectric mode primarily detected time‐varying loading and unloading events. The following section evaluates the stretch‐induced strain‐insensitive pressure sensing performance.

## 4 Validation of Stretch‐Induced Strain‐Insensitive Pressure Sensing Performance

### 4.1 Effect of Arc Opening Angle on Strain Accommodation

Calibration curves were measured under different stretching ratios. Stretch‐induced strain‐insensitive pressure sensing performance was quantified by comparing variations among these calibration curves using average capacitance/piezoelectric output, standard deviation, and sensitivity. Under different stretching ratios, deviations among calibration curves originated from coupling between in‐plane stretching and out‐of‐plane deformation within the core sensing layer [49]. Geometric parameters of the *m*‐MEX printed TPU 3D architecture governed how effectively stretch‐induced strain was accommodated. A serpentine unit cell consisted of two curved sections (arcs) and three straight segments (arms) [49]. The in‐plane geometry was defined by three independent parameters: arc radius (*R*), width (*w*), and arc opening angle (*α*). Among these parameters, the effect of arc opening angle (*α*) was investigated while other parameters remained constant.

**FIGURE 2:**
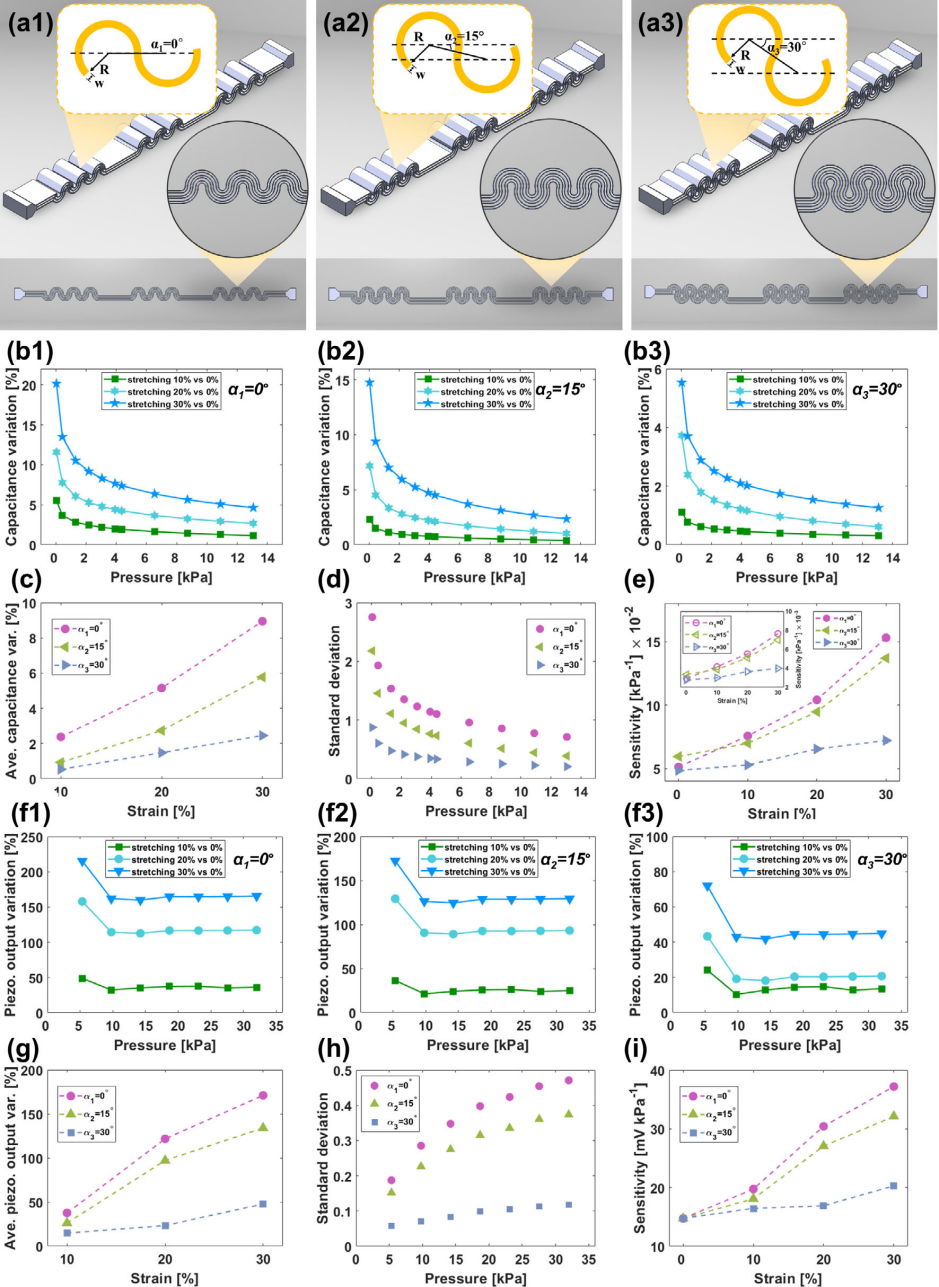
Validation of stretch‐induced strain‐insensitive sensing performance through arc‐opening‐angle control of the serpentine architecture. (a) CAD models of serpentine architectures with arc opening angles *α* = 0°, 15°, and 30°, fabricated by m‐MEX, illustrating the in‐plane geometric parameters: arc radius (*R*), width (*w*), and arc opening angle (*α*). (b) Capacitance–pressure calibration curve variations measured at applied strains of 0%, 10%, 20%, and 30%. (c) Average capacitance variation over the full pressure range (0–14 kPa) at applied strains of 0%, 10%, 20%, and 30%. (d) Standard deviation of capacitance–pressure calibration curves. (e) Capacitive sensitivity of devices with *α* = 0°, 15°, and 30° at applied strains of 0%, 10%, 20%, and 30%. (f) Piezoelectric output–pressure calibration curve variations measured at applied strains of 0%, 10%, 20%, and 30%. (g) Average piezoelectric output variation at applied strains of 0%, 10%, 20%, and 30%. (h) Standard deviation of piezoelectric output–pressure calibration curves. (i) Piezoelectric sensitivity of devices with *α* = 0°, 15°, and 30° at applied strains of 0%, 10%, 20%, and 30%.

Three arc opening angles (*α_1_* = 0°, *α_2_* = 15°, and *α_3_* = 30°) were selected, with the corresponding computer‐aided design (CAD) models shown in Figure 2a. In the *m*‐MEX printed architectures, arc opening angles exceeding 30° could not be fabricated reproducibly. At larger arc opening angles, the curvature of adjacent serpentine segments approached the nozzle diameter, resulting in unstable extrusion paths, filament buckling, and loss of dimensional accuracy. This printing constraint defined the upper limit of arc opening angles achievable with the current fabrication process.

Capacitive pressure sensors with three arc opening angles were evaluated to examine the influence of structural geometry on stretch‐induced interference. Capacitance–pressure curves were measured under applied uniaxial strains of 0%, 10%, 20%, and 30% for sensors with different arc opening angles (shown in Figure S4). For each design, stretch‐induced capacitance variation was obtained by normalizing the capacitance–pressure curves measured under applied strain to the corresponding response at 0% strain, yielding percentage variation as a function of pressure (Figure 2b). For a given arc opening angle, larger capacitance variations were observed in the low‐pressure range (0–2 kPa). As pressure increased beyond 2 kPa, the variation decreased and gradually stabilized up to 14 kPa. This pressure‐dependent trend was consistently observed across all applied strain levels. Increasing the applied strain led to a monotonic increase in capacitance variation, reflecting increased mechanical perturbation of the sensing architecture.

For an ideal strain‐insensitive pressure sensor, capacitance depended solely on applied pressure and remained independent of the stretching ratio. Experimental results indicated that the initial capacitance at 0 kPa decreased with increasing stretching ratio because tensile strain (*ξ*) altered the effective electrode area *A(ξ)* and dielectric thickness *d(ξ)*. According to the fundamental capacitance relationship *C*  =  ε*A*/*d*, the pressure‐dependent contribution arose from coupled variations in dielectric constant *ε(ξ)* and the geometric factor *A(ξ)/d(ξ)*. Increasing applied pressure compressed the dielectric layer, reduced *d(ξ)*, and slightly increased *ε(ξ)*, leading to an overall increase in capacitance. When stretching was applied, spatially non‐uniform variations in effective electrode area and dielectric thickness generated deviations among calibration curves.

These results indicated incomplete accommodation of stretch‐induced strain by the serpentine architecture, with a portion of the strain absorbed through elastic deformation within the planar sensing region. From a mechanistic perspective, the high dielectric constant of the PVdF/TPU composite microfibers enhanced charge‐storage capability, while the porous electrospun morphology increased effective electrode contact area. In addition, the low Young's modulus introduced by TPU enabled substantial dielectric compression under small applied pressures, thereby amplifying capacitance variation and enhancing sensitivity in the low‐pressure range (0–2 kPa).

A comparison across different arc opening angles revealed a pronounced geometry‐dependent effect. Under identical strain conditions, devices with larger arc opening angles exhibited reduced capacitance variation across the entire pressure range. This reduction indicated that increasing the arc opening angle allowed a greater portion of the stretch‐induced strain to be accommodated by the 3D serpentine architecture, thereby limiting strain transfer to the capacitive sensing layer. At *α_1_* = 0°, the serpentine arms provided less geometric freedom for in‐plane rotation, causing a larger fraction of the applied strain to be transferred to the planar sensing region. This strain transfer produced greater changes in the effective electrode area, dielectric thickness, and local contact configuration, resulting in the largest stretch‐induced capacitance variation. In contrast, the *α_3_* = 30° architecture provided greater rotational compliance and accommodated more of the applied strain within the serpentine region, thereby minimizing deformation of the dielectric layer and producing the smallest capacitance variation.

To quantify this effect, the average capacitance variation was calculated over the full pressure range for each design and plotted as a function of applied strain (Figure 2c). For *α_1_* = 0°, the average variation increased from 2.4% at 10% strain to 5.2% at 20% strain and 8.9% at 30% strain. In comparison, devices with *α_2_* = 15° exhibited reduced average variations of 0.9%, 2.7%, and 5.8% under the same strain levels. The *α_3_* = 30° design showed the smallest variations, limited to 0.5%, 1.5%, and 2.4% at 10%, 20%, and 30% strain, respectively. At 10% strain, increasing the arc opening angle from 0° to 15° and 30° reduced the average capacitance variation by 62.5% and 79.2%, respectively. Corresponding reductions of 48.1% and 71.2% were observed at 20% strain, and 34.8% and 73.0% at 30% strain. These results established the arc opening angle as an effective structural parameter for suppressing stretch‐induced interference in capacitive pressure sensing. At 30% strain, the *α_3_* = 30° design achieved a 73.0% reduction in average capacitance variation relative to the *α_1_* = 0° design.

Stretch‐induced capacitance interference was further evaluated by calculating the standard deviation at fixed applied pressures (Figure 2d). The largest standard deviations occurred in the low‐pressure range (0–2 kPa) and decreased with increasing pressure, as the pressure‐dependent capacitance component became dominant, thereby reducing the relative impact of stretch‐induced interference. Increasing the arc opening angle from 0° to 15° and 30° reduced the average standard deviation from 1.30 to 0.91 and 0.40, respectively, indicating progressively improved strain accommodation.

Stretch‐induced strain‐dependent changes in capacitive sensitivity were also examined (Figure 2e), with sensitivity extracted separately in the low‐pressure (0–2 kPa) and high‐pressure (2–14 kPa) ranges. For a fixed geometry, sensitivity increased with applied strain in both ranges; however, the magnitude of this increase decreased with increasing arc opening angle. For devices with *α_1_* = 0°, sensitivity increased from 5.1 × 10^−2^ to 1.5 × 10^−1^ kPa^−1^ (194.1%) in the 0–2 kPa range and from 2.9 × 10^−3^ to 7.6 × 10^−3^ kPa^−1^ (162.1%) in the 2–14 kPa range as strain increased from 0% to 30%. For *α_2_* = 15°, sensitivity increased from 6.0 × 10^−2^ to 1.4 × 10^−1^ kPa^−1^ (133.3%) in the 0–2 kPa range and from 3.3 × 10^−3^ to 7.0 × 10^−3^ kPa^−1^ (112.1%) in the 2–14 kPa range over the same strain range. In contrast, devices with α_3_ = 30° exhibited substantially reduced strain dependence, with sensitivity increasing from 4.9 × 10^−2^ to 7.2 × 10^−2^ kPa^−1^ (46.9%) in the 0–2 kPa range and from 2.8 × 10^−3^ to 4.0 × 10^−3^ kPa^−1^ (42.9%) in the 2–14 kPa range. These results indicated that increasing the arc opening angle effectively suppressed stretch‐induced variations in capacitive sensitivity, with the *α_3_* = 30° architecture exhibiting the highest robustness under applied strain.

The effect of arc opening angle on stretch‐induced interference was further investigated under piezoelectric sensing mode. Devices with *α_1_* = 0°, *α_2_* = 15°, and *α_3_* = 30° were tested by measuring piezoelectric output as a function of applied pressure under strains of 0%, 10%, 20%, and 30%. Piezoelectric output voltage recorded under stepwise increasing pressure, and piezoelectric output–pressure calibration curves measured at different applied strains for sensors with different arc opening angles are shown in Figure S4. Percentage piezoelectric output variation was calculated relative to the 0% strain response, yielding the stretch‐induced variation profiles shown in Figure 2f. For all designs, larger piezoelectric output variations occurred in the low‐pressure range (0–5 kPa). As pressure increased beyond 5 kPa, the variation decreased and stabilized up to 35 kPa, as the pressure‐dependent piezoelectric response dominated over stretch‐induced perturbations. Increasing applied strain led to increased variation across the full pressure range (0–35 kPa). At a fixed strain level, however, increasing the arc opening angle systematically reduced the magnitude of piezoelectric output variation. At *α_1_* = 0°, limited rotational deformation of the serpentine architecture allowed more tensile strain to reach the electrospun PVdF/TPU sensing layer. The resulting changes in microfiber deformation, local stress distribution, and electrode‐sensing‐layer contact produced the largest stretch‐induced piezoelectric output variation. Increasing the arc opening angle enhanced the geometric compliance of the serpentine structure and reduced strain transfer to the active piezoelectric region. Therefore, the *α_3_* = 30° design produced the smallest stretch‐induced piezoelectric output variation.

Average piezoelectric output variations were calculated to enable quantitative comparison (Figure 2g). For *α_1_* = 0°, the average variation increased from 37.6% at 10% strain to 121.8% at 20% strain and 171.1% at 30% strain. Devices with *α_2_* = 15° exhibited lower variations of 26.1%, 97.1%, and 134.1%, while *α_3_* = 30° devices showed substantially reduced values of 14.6%, 23.2%, and 47.8%, respectively. At 10% strain, increasing the arc opening angle from 0° to 15° and 30° reduced the average piezoelectric output variation by 30.5% and 61.0%, respectively. At 20% strain, the corresponding reductions were 20.2% and 80.9%, and at 30% strain they reached 21.6% and 72.0%. These results indicated that increasing arc opening angles significantly suppressed stretch‐induced perturbations in piezoelectric output. At 30% strain, the *α_3_* = 30° design achieved a 72.0% reduction in average variation relative to the *α_1_* = 0° design. Standard deviation analysis further supported this trend (Figure 2h). Increasing the arc opening angle from 0° to 15° and 30° reduced the average standard deviation from 0.37 to 0.29 and 0.09, respectively.

Piezoelectric sensitivity was extracted by linear regression of output versus pressure (Figure 2i). For *α_1_* = 0°, sensitivity increased from 14.7 to 37.2 mV kPa^−1^ as strain increased from 0% to 30%, corresponding to a 153.1% increase. Devices with *α_2_* = 15° showed a reduced increase of 118.4%. In contrast, *α_3_* = 30° exhibited a sensitivity increase of only 38.1% over the same strain range, indicating substantially reduced strain dependence.

Collectively, these results highlighted the effectiveness of arc‐engineered 3D architectures for enabling strain‐insensitive multimodal pressure sensing. Under capacitive sensing mode, the *α_3_* = 30° architecture maintained approximately 97.6% strain insensitivity, as defined by capacitance variation, up to 30% applied strain and achieved a minimum detectable pressure of 0.04 kPa, supporting reliable static pressure detection. Under piezoelectric sensing mode, the same architecture provided a sensitivity of 16.5 mV kPa^−1^ with a strain‐insensitivity ratio of 85.4% at 10% strain. At 30% strain, a sensitivity of 20.3 mV kPa^−1^ was retained, corresponding to a strain‐insensitivity ratio of 52.2%.

### 4.2 Effect of Center‐Layer Thickness on Strain‐Insensitive Sensing

**FIGURE 3:**
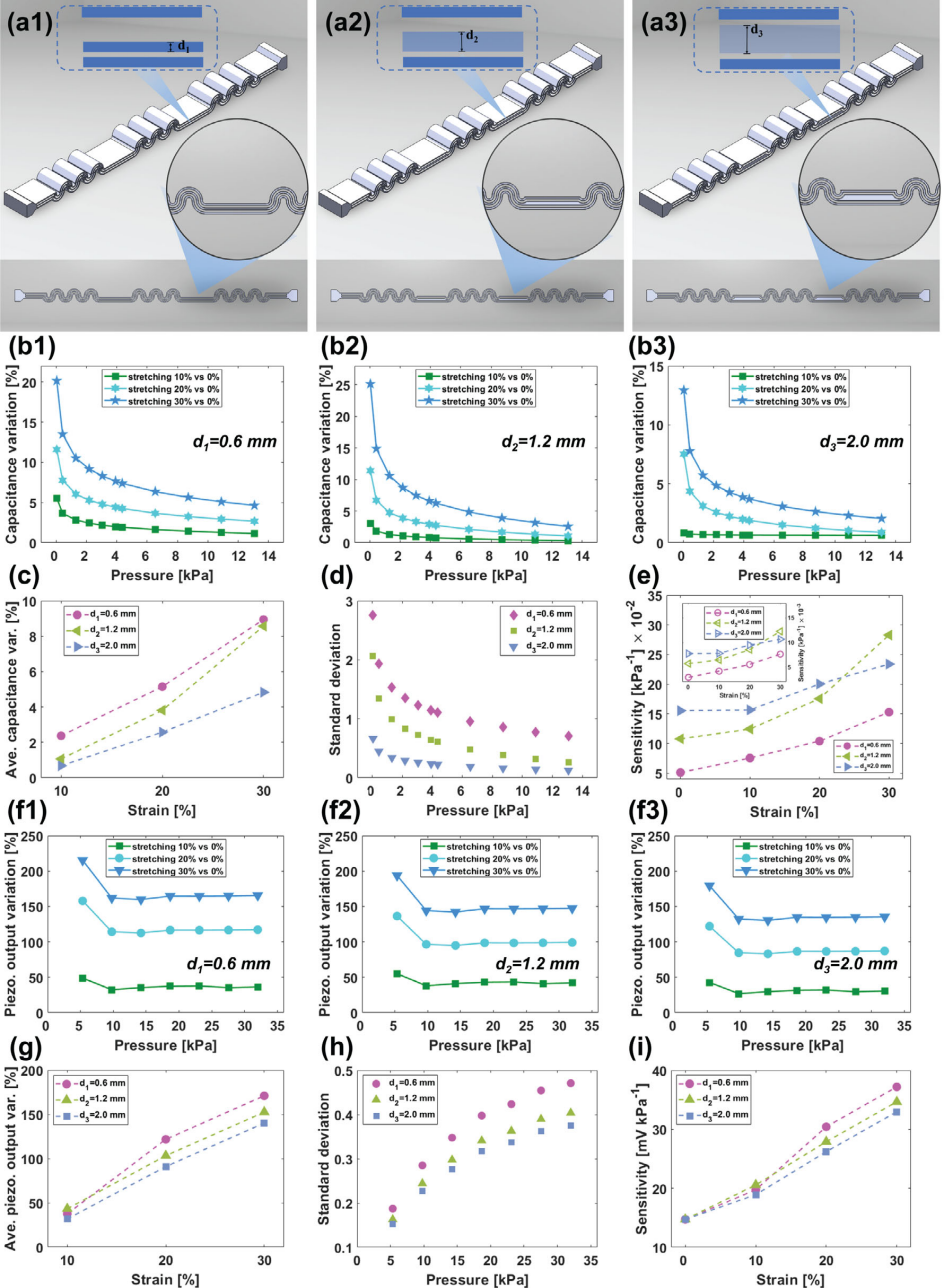
Validation of stretch‐induced strain‐insensitive sensing performance through center‐layer thickness control of the serpentine architecture. (a) CAD models of serpentine structures with center‐layer thicknesses *d* = 0.6, 1.2, and 2.0 mm. (b) Capacitance–pressure calibration curve variations at applied strains of 0%, 10%, 20%, and 30%. (c) Average capacitance variation over the full pressure range (0–14 kPa) measured at applied strains of 0%, 10%, 20%, and 30%. (d) Standard deviation of capacitance–pressure calibration curves. (e) Capacitive sensitivity of devices with center‐layer thicknesses *d* = 0.6, 1.2, and 2.0 mm at applied strains of 0%, 10%, 20%, and 30%. (f) Piezoelectric output–pressure calibration curve variations at applied strains of 0%, 10%, 20%, and 30%. (g) Average piezoelectric output variation at applied strains of 0%, 10%, 20%, and 30%. (h) Standard deviation of piezoelectric output–pressure calibration curves. (i) Piezoelectric sensitivity of devices with center‐layer thicknesses *d* = 0.6, 1.2, and 2.0 mm at applied strains of 0%, 10%, 20%, and 30%.

The influence of center‐layer thickness in the *m*‐MEX printed 3D architecture on sensing performance was systematically investigated (Figure 3). Three center‐layer thicknesses (*d_1_* = 0.6 mm, *d_2_* = 1.2 mm, and *d_3_* = 2.0 mm) were designed, and the corresponding CAD models are presented in Figure 3a. All other geometric parameters of the architecture remained constant. Capacitance–pressure calibration curves for sensors with different center‐layer thicknesses measured at different applied strains are shown in Figure S5.

Increasing center‐layer thickness increased the overall rigidity of the TPU architecture, redistributing strain energy away from the active sensing region. The thicker central layer constrained lateral deformation, thereby minimizing tensile distortion of the dielectric layer and stabilizing capacitance under applied strain. This improvement arose from a more uniform deformation field and a reduced vertical compliance gradient, which collectively suppressed non‐uniform strain localization within the sensing layer.

To assess the influence of the center‐layer thickness on stretch‐induced interference, capacitive sensors with three center‐layer thicknesses (*d_1_* = 0.6 mm, *d_2_* = 1.2 mm, and *d_3_* = 2.0 mm) were characterized. Capacitance–pressure responses were measured under applied strains of 0%, 10%, 20%, and 30%. The strain‐induced capacitance variation was calculated by normalizing the capacitance–pressure curves measured at 10%, 20%, and 30% strain to the corresponding 0% strain response, yielding the percentage variation as a function of applied pressure (Figure 3b). For each thickness, the largest capacitance variations occurred in the low‐pressure range (0–2 kPa). As pressure increased to 2–14 kPa, the variation decreased and approached a steady level. This pressure‐dependent trend persisted across all strain conditions, while the magnitude of variation increased with increasing applied strain. When comparing different thicknesses at the same strain, increasing center‐layer thickness reduced capacitance variation across the entire pressure range (0–14 kPa), indicating that a thicker center layer better suppressed strain transfer to the capacitive sensing region.

The average capacitance variations, calculated over the full pressure range, are summarized in Figure 3c. For *d_1_* = 0.6 mm, the average variation increased from 2.4% (10% strain) to 5.2% (20% strain) and 8.9% (30% strain). For *d_2_* = 1.2 mm, the corresponding values were 1.1%, 3.8%, and 8.6%. For *d_3_* = 2.0 mm, the average variation was further reduced to 0.7%, 2.6%, and 4.8% at 10%, 20%, and 30% strain, respectively. At 10% strain, increasing thickness from 0.6 mm to 1.2 mm and 2.0 mm reduced the average capacitance variation by 54.2% and 70.8%, respectively. At 20% strain, the reductions were 26.9% and 50.0%, and at 30% strain they reached 3.4% and 46.1%, respectively. Under 30% strain, increasing the center‐layer thickness from 0.6 mm to 2.0 mm decreased the average capacitance variation by 46.1%, confirming the benefit of thickness optimization for strain‐insensitive capacitive sensing.

Stretch‐induced interference in capacitance sensing was further evaluated by calculating the standard deviation at fixed applied pressures (Figure 3d). The largest standard deviations occurred at 0–2 kPa and decreased with increasing pressure, consistent with the reduced relative contribution of strain‐induced perturbations at higher pressures, where the pressure‐dependent capacitance component dominated. Increasing thickness from *d_1_* = 0.6 mm to *d_2_* = 1.2 mm and *d_3_* = 2.0 mm reduced the average standard deviation from 1.30 to 0.79 and 0.28, respectively, indicating improved stability of capacitance readings under deformation for thicker center layers.

Stretch‐induced interference in sensitivity was also analyzed (Figure 3e), with sensitivity extracted separately in the 0–2 kPa and 2–14 kPa pressure ranges. For each thickness, sensitivity increased with applied strain; however, the strain dependence decreased as the center layer became thicker. For *d_1_* = 0.6 mm, sensitivity increased from 5.1 × 10^−2^ to 1.5 × 10^−1^ kPa^−1^ (194.1%) in the 0–2 kPa range and from 2.9 × 10^−3^ to 7.6 × 10^−3^ kPa^−1^ (162.1%) in the 2–14 kPa range as strain increased from 0% to 30%. For *d_2_* = 1.2 mm, sensitivity increased from 1.1 × 10^−1^ to 2.8 × 10^−1^ kPa^−1^ (154.5%) and from 5.7 × 10^−3^ to 1.2 × 10^−2^ kPa^−1^ (110.5%) in the two ranges, respectively. For *d_3_* = 2.0 mm, sensitivity changes were substantially smaller, increasing from 1.6 × 10^−1^ to 2.3 × 10^−1^ kPa^−1^ (43.8%) in the 0–2 kPa range and from 7.7 × 10^−3^ to 1.1 × 10^−2^ kPa^−1^ (42.9%) in the 2–14 kPa range. These results indicated that increasing the center‐layer thickness reduced the strain‐induced shift in capacitive sensitivity.

Piezoelectric outputs were measured for devices with *d_1_* = 0.6 mm, *d_2_* = 1.2 mm, and *d_3_* = 2.0 mm under applied strains of 0%, 10%, 20%, and 30%. Piezoelectric output voltage was recorded under stepwise increasing pressure, and piezoelectric output–pressure calibration curves for sensors with different center‐layer thicknesses measured at different applied strains are shown in Figure S5. Percentage output variation was calculated relative to the 0% strain response to obtain strain‐induced variation profiles as a function of applied pressure (Figure 3f). For each thickness, the largest piezoelectric output variations were observed at low pressures (0–5 kPa), followed by reduced and stabilized variations at higher pressures (5–35 kPa). Output variation increased with increasing applied strain for all devices. At a fixed strain condition, thicker center layers generally reduced the magnitude of output variation across the full pressure range, although the effect was less pronounced than in the capacitive mode.

Average piezoelectric output variation is summarized in Figure 3g. For *d_1_* = 0.6 mm, the average variation increased from 37.6% (10% strain) to 121.8% (20% strain) and 171.1% (30% strain). For *d_2_* = 1.2 mm, the corresponding values were 43.3%, 103.3%, and 152.6%. For *d_3_* = 2.0 mm, the values were 31.8%, 91.0%, and 140.3%. At 10% strain, the *d_2_* = 1.2 mm device showed a higher average variation than the *d_1_* = 0.6 mm device, while the *d_3_* = 2.0 mm device reduced the variation by 15.3% relative to the *d_1_* = 0.6 mm device. At 20% strain, the average variation decreased by 15.2% (*d_2_*) and 25.3% (*d_3_*) relative to the *d_1_* device. At 30% strain, the reductions were 10.8% (*d_2_*) and 18.0% (*d_3_*). A nonmonotonic piezoelectric output variation was observed only at 10% strain, where the *d_2_* = 1.2 mm device showed a larger variation than the *d_1_* = 0.6 mm device, while the *d_3_* = 2.0 mm device showed the smallest variation. The thinner *d_1_* structure was more compliant and may have accommodated small tensile deformation through more uniform global deformation of the soft architecture, thereby reducing the change in local pressure‐induced deformation of the electrospun PVdF/TPU microfiber layer between the unstretched and stretched states. In contrast, the *d_2_* structure introduced an intermediate constraint that was not sufficient to fully isolate the active piezoelectric layer but may have redistributed the applied tensile strain into more localized deformation near the sensing region, resulting in a larger piezoelectric output variation. When the center‐layer thickness was further increased to *d_3_* = 2.0 mm, the structural constraint became stronger, reducing strain transfer to the active sensing region and producing the smallest piezoelectric output variation at 10% strain. At 20% and higher applied strains, the structural constraint provided by increasing center‐layer thickness became dominant, resulting in reduced piezoelectric output variation. Overall, increasing thickness to *d_3_* = 2.0 mm decreased the average piezoelectric output variation by 18.0% at 30% strain.

Standard deviation analysis (Figure 3h) showed the largest variation at low pressure (0–5 kPa), followed by a decrease with increasing pressure. Increasing thickness from *d_1_* to *d_2_* and *d_3_* reduced the average standard deviation from 0.37 to 0.31 and 0.29, respectively, indicating a modest improvement in the stability of piezoelectric output with increased thickness. Piezoelectric sensitivity was extracted by linear regression of piezoelectric output versus applied pressure (Figure 3i). For *d_1_* = 0.6 mm, sensitivity increased from 14.7 to 37.2 mV kPa^−1^ as strain increased from 0% to 30% (153.1%). For *d_2_* = 1.2 mm, sensitivity increased from 14.7 to 34.7 mV kPa^−1^ (136.1%). For *d_3_* = 2.0 mm, sensitivity increased from 14.7 to 33.0 mV kPa^−1^ (124.5%). These results indicated that increasing center‐layer thickness reduced stretch‐induced sensitivity drift, but the improvement remained moderate compared with the capacitive case.

Increasing center‐layer thickness improved mechanical stability and reduced stretch‐induced signal interference, with a stronger benefit observed for capacitive sensing than for piezoelectric sensing. Under the capacitive sensing mode, the optimized sensor with *d_3_* = 2.0 mm exhibited approximately 95.2% strain insensitivity up to 30% applied strain and a minimum detectable pressure of 0.04 kPa, supporting static pressure detection. Under the piezoelectric sensing mode, the same device exhibited a sensitivity of 18.9 mV kPa^−1^ with a strain‐insensitivity ratio of 68.2% at 10% strain. At 20% strain, the device maintained a sensitivity of 26.2 mV kPa^−1^ with a strain‐insensitivity ratio of 9.0%. These results demonstrated that tuning the arc opening angle and controlling the center‐layer thickness played critical roles in achieving stretch‐induced strain‐insensitive pressure sensing in both capacitive and piezoelectric modes.

The manufacturing method and sensing performance of the present sensor are compared with recent top‐tier flexible pressure sensors in Table S12. The dynamic durability of the sensor (*α_3_* = 30°) was further evaluated through cyclic press‐release testing and 5,000 tensile‐release cycles from 0% to 30% strain, with the corresponding piezoelectric pressure‐sensing performance before and after fatigue treatment provided in Section S5. An analytical model describing the mechanics of the island‐bridge serpentine configuration and its influence on *stretchability* and *effective stiffness* is presented in the Discussion section.

## 5 Fully Additively Manufactured Stretchable Capacitive Sensing Matrix for Pressure Mapping

To demonstrate the application of the flexible and stretchable pressure sensor in pressure mapping, a stretchable 4 × 4 capacitive sensing matrix was constructed, as shown in Figure S9. The electrospun core sensing layer specifically designed for the stretchable 4 × 4 sensing matrix is shown in Figure S9a. The soft 3D architecture was redesigned such that each *m*‐MEX printed TPU architecture integrated four individual sensors and functioned as a single sensing module (1 × 4 matrix), as shown in Figure S9b. Stretchability provided by the serpentine architecture ensured that externally applied mechanical deformation, including bending and stretching, did not alter the capacitance–pressure calibration curves. Stable sensing performance was maintained as the center‐to‐center distance between adjacent sensors varied under applied strains ranging from 0% to 30%.

The scale of the 1 × 4 sensing matrix was expanded by adding additional rows to build a 2 × 4 sensing matrix, as shown in Figure S9c. In addition, gaps between rows provided intrinsic mechanical isolation, effectively suppressing crosstalk. This scalable matrix design enabled independent deformation of each sensing element without mutual interference, allowing reliable operation under stretching or bending during practical use. Three static weights were applied to three different sensing elements. Each applied weight produced a distinct capacitance increase proportional to the applied pressure. Capacitance variations of all eight sensors in the 2 × 4 sensing matrix configuration were measured and recorded, with the results presented in Figure S9d.

The matrix was subsequently expanded to a 4 × 4 sensing matrix, as shown in Figure S9e. Three static weights were placed on sensors located at different positions within the array. The capacitance of each of the sixteen sensors was measured independently, ensuring consistent, decoupled readout across the sensing matrix. As shown in Figure S9f, sensors subjected to applied loads exhibited capacitance increases of approximately 8%–12% relative to baseline, while unloaded sensors displayed negligible drift of less than 0.5%. Localized capacitance peaks at loaded positions confirm spatial selectivity and minimal mechanical and electrical interference between neighboring sensing elements, validating accurate pressure‐mapping performance. The sensing‐matrix architecture supported scaling, enabling coverage of larger surface areas while preserving mechanical compliance and sensing precision without repeating the full design–fabrication–testing cycle.

## 6 Proof‐of‐Concept Demonstration in a Soft Gripper Application

**FIGURE 4:**
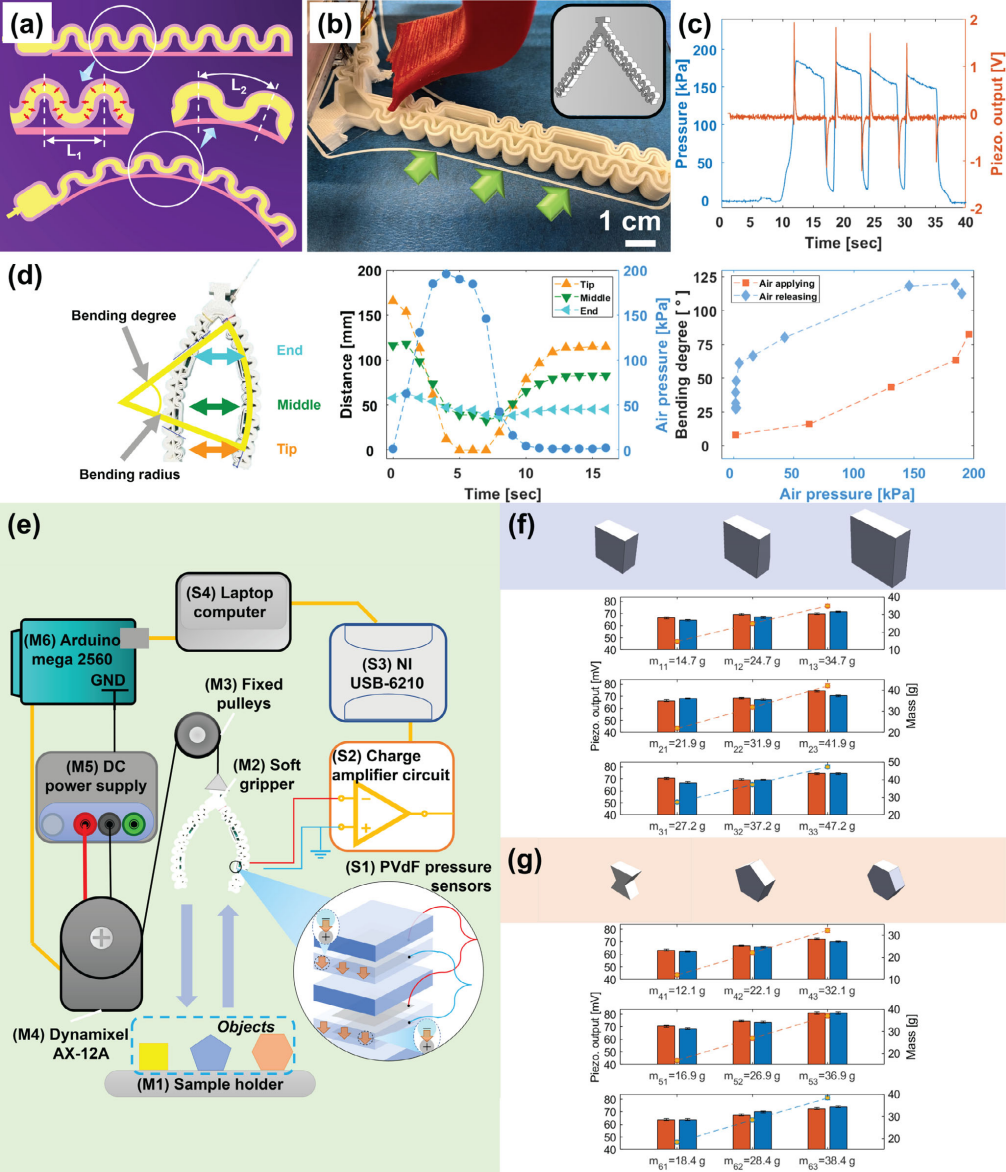
Integration of stretchable piezoelectric pressure sensors within a pneumatic soft gripper. (a) Schematic illustration of the operating principle of the pneumatic soft actuator, showing differential expansion between the outer and inner walls during inflation, which drives inward bending. (b) *m*‐MEX printing of a soft gripper with integrated 3D architectures for stretchable pressure sensors. (c) Time‐resolved air pressure and piezoelectric voltage output during multiple grasp–release cycles. (d) Measured deformation of the gripper fingers at three characteristic positions (“Tip,” “Middle,” and “End”) as a function of air pressure. (e) Experimental setup for actuation and data acquisition. Piezoelectric output for grasping objects of various (f) masses, and (g) geometries.

The combination of multiple additive manufacturing techniques enabled co‐fabrication of flexible and stretchable pressure sensors that could be directly integrated into soft robotic actuators during device fabrication [43]. As a proof‐of‐concept demonstration, a fully *m*‐MEX printed soft gripper was developed with a pair of flexible and stretchable pressure sensors embedded within the contact regions between the soft gripper and rigid objects, as illustrated in Figure 4. Grasping motion of the soft gripper was pneumatically actuated, as schematically shown in Figure 4a. Increasing internal air pressure generated a differential extension between the outer and inner sides of the actuator, producing inward bending that enabled grasping. The soft 3D architecture of both the stretchable sensor and the actuator was additively manufactured in a single *m*‐MEX printing process, as shown in Figure 4b, with an inset presenting a CAD model of the designed soft gripper. Conformal gripper interfaces formed through direct additive integration preserved actuator compliance and eliminated delamination commonly observed in post‐attached sensors. Following printing, core sensing layers were integrated with electrical wiring connected to signal‐conditioning circuitry to complete the device fabrication.

A representative piezoelectric output voltage recorded from the integrated sensor, together with the corresponding pneumatic pressure during operation, is shown in Figure 4c. In this proof‐of‐concept demonstration, only the piezoelectric mode was used to detect dynamic contact, grasping, and releasing events. The capacitive mode was not used to quantify gripping pressure. Owing to the flexibility of the electrospun core sensing layer and the strain‐accommodating serpentine architecture, pneumatic actuation alone generated minimal piezoelectric output. In contrast, object contact produced a pronounced piezoelectric response, confirming that the dominant signal originated from contact‐induced pressure. The piezoelectric signal accurately reflected dynamic contact behavior without interference from actuator bending or stretching. During inflation associated with object grasping, increased air pressure induced inward bending of the actuator and contact with the object, producing a positive voltage spike. During deflation corresponding to object release, decreased air pressure reduced the contact force, generating a negative voltage spike as the contact‐induced pressure diminished. Temporal alignment among piezoelectric voltage spikes, pneumatic pressure profiles, and recorded kinematic deformation confirmed real‐time sensing of grasping and releasing events. These results demonstrated that the integrated stretchable piezoelectric pressure sensors effectively suppressed interference arising from actuator deformation while maintaining accurate detection of object‐contact‐induced pressure. Polarity‐specific voltage signatures corresponding to grasp and release events could enable closed‐loop feedback control for adaptive manipulation. Compared with conventionally attached sensors, which cannot distinguish air‐pressure‐driven deformation from contact‐force‐induced pressure, the all‐additive manufacturing technique ensured conformal integration, preserved actuator compliance, and simplified fabrication. The grasping and releasing process of the soft gripper is further demonstrated in Movie S1.

Geometric parameters used to quantify actuator deformation included the bending angle, as defined and illustrated in Figure 4d. Three representative positions between the two finger‐shaped actuators were designated as “Tip,” “Middle,” and “End.” During a typical grasp–release cycle, the geometric deformation of the soft gripper as a function of applied pneumatic pressure was recorded and plotted in Figure 4d. Time‐resolved variations in the distances between the two actuators at these three locations, together with the applied air pressure, were also plotted. The distances between the actuators decreased monotonically with increasing air pressure. The “Tip” location exhibited the largest displacement, indicating dominant participation in the grasping process and identifying it as the optimal sensing location. As the pneumatic pressure increased from 0 kPa to 198 kPa, the distance between the two tips decreased from 162 mm to 0 mm, corresponding to complete closure of the gripper. A small response delay was observed: the air pressure reached its maximum at 4.5 s, whereas the tip distance reached 0 mm at 5.0 s. This delay formed a mechanical hysteresis loop associated with the viscoelastic response of the pneumatic actuator. Despite this delay, the integrated piezoelectric sensors generated immediate voltage spikes that clearly delineated contact and release timing. Placement of the sensor at the tip concentrated mechanical contact‐induced pressure within the electrospun microfiber network, thereby enhancing pressure‐to‐charge conversion efficiency and improving the signal‐to‐noise ratio. The electromechanical hysteresis of the piezoelectric response during cyclic loading and unloading was further quantified using displacement‐dependent hysteresis curves, as provided in Figure S10, Section S7

A control system was developed to manipulate the soft gripper and acquire corresponding piezoelectric signals during operation, as shown in Figure 4e. The soft gripper was evaluated by grasping square‐shaped rigid objects of different sizes and masses. Average peak piezoelectric output amplitudes measured from the two embedded pressure sensors within the soft gripper fingers were extracted and plotted as paired bar graphs for objects with varying mass (Figure 4f). The signals from the two fingers were distinguished by different colors. When grasping objects with different geometries, the bending radii of the pneumatic fingers and the inter‐finger distances at representative contact locations differed, indicating geometry‐dependent deformation of the compliant soft gripper structure. During lifting, static equilibrium required that the sum of the contact and friction forces exerted by the two fingers balance the gravitational force of the object. However, the embedded sensors measured local contact force‐induced pressure rather than total object weight. The normal force (*F_n_*) transmitted to the sensing layer depended on the contact angle (θ) between the normal‐force direction and the direction of the object’s weight (*W*), according to $W=F_{n}\;\cos\left(\theta \right)$. Moreover, the measured pressure was governed by *P*  = *F_n_*/*A_c_* , where *A_c_* denotes the effective contact area. Because both the contact angles (θ) and the effective contact area (*A_c_*) varied with object geometry and finger deformation, objects of identical mass could produce different local force distributions. Therefore, a universal calibration directly relating piezoelectric output to object weight was not physically expected. Instead, the measured voltage reflected geometry‐dependent local contact force‐induced pressure within the compliant soft gripper structure. Consistent voltage outputs across repeated trials confirmed the reproducibility and robustness of the integrated pressure‐sensing system. Additional experiments involving objects with different geometries yielded comparable pressure–response trends (Figure 4g), demonstrating reliable conformal grasping behavior and stable pressure sensing under deformation.

## 7 Discussion

To overcome the limited stretchability of the electrospun sensing layer, an *m*‐MEX printed serpentine architecture was introduced to achieve system‐level stretchability by reconfiguring the electrospun microfiber membrane into a compliant 3D serpentine geometry. From an engineering design standpoint, in this sensor architecture, the active sensing components were positioned on discrete “islands” that were mechanically and electrically connected through conductive, stretchable serpentine interconnects. Stretch‐induced strain in the device was primarily accommodated by the serpentine region. The serpentine thickness typically exceeded its width, and the high stretchability primarily arose from the in‐plane rotational deformation of the serpentine arms.

*Stretchability* and *effective stiffness* are particularly significant mechanical characteristics of serpentine structures. The *stretchability* of a serpentine structure, denoted as $\varepsilon_{app}^{cr}$, is defined as the maximum macroscopic applied strain that the structure can sustain before material failure occurs locally and is expressed as:

(1) $$\varepsilon_{app}^{cr}=\frac{\varepsilon_{cr}}{\varepsilon_{max}/\varepsilon_{app}}\;$$

Failure is assumed to be initiated when the maximum tensile strain within the serpentine reaches the intrinsic strain‐to‐rupture of the constituent material, ε_*cr*_. The normalized maximum tensile strain in the serpentine, ε_*max*_/ε_*app*_, represents the geometric strain amplification factor, where ε_*max*_is the maximum local tensile strain within the serpentine and ε_*app*_is the applied macroscopic strain. The normalized maximum tensile strain in the serpentine ε_*max*_/ε_*app*_ as a dimensionless quantity depends solely on the serpentine geometry and is independent of material properties under linear elastic conditions. Serpentine geometries that minimize ε_*max*_/ε_*app*_ effectively decouple global deformation from local material strain, enabling brittle materials with low intrinsic failure strains to accommodate applied deformations. This *stretchability* expression directly links material failure limits to geometry‐driven strain redistribution. This framework provides a theoretical foundation for optimizing serpentine geometry designs to achieve enhanced *stretchability* without altering material composition.

*Effective stiffness, k_eff_*, is defined as the ratio of the reaction force (*F_0_*) to the effective displacement (2*δ_0_*). The *effective stiffness*–geometry relationships were derived analytically under linear elastic and plane‐strain conditions using curved‐beam theory, as reported in the literature. Detailed derivations are provided in Section S8. The *effective stiffness* is expressed as:

(2) $$k_{eff}=k_{eff,2}+\frac{4k_{eff,1}k_{eff,3}}{k_{eff,1}+k_{eff,3}}$$

The corresponding *effective stiffnesses* of the three laminated constituent unit cells are denoted *k*_*eff*,1_, *k*_*eff*,2_, and *k*_*eff*,3_, respectively. This analytical framework enables optimization of these parameters to achieve maximum *stretchability* and minimum *effective stiffness* while satisfying two essential constraints: prevention of material overlap and adherence to the finite structural width imposed by the fabrication resolution.

## 8 Conclusion

Stretch‐induced strain‐insensitive pressure sensing was achieved by integrating the electrospun sensing layer with a 3D strain‐accommodating architecture that reconfigured the sensing layer into a stretchable serpentine geometry. In the capacitive sensing mode, the optimized design with an arc opening angle of *α_3_* = 30° achieved approximately 97.6% strain insensitivity at 30% applied strain, with a minimum detectable pressure of 0.04 kPa. In the piezoelectric sensing mode, the same device achieved a dynamic sensitivity of 16.5 mV kPa^−1^ with a strain‐insensitivity ratio of 85.4% at 10% applied strain. At 30% applied strain, the device maintained a sensitivity of 20.3 mV kPa^−1^ with a strain‐insensitivity ratio of 52.2%. A stretchable capacitive sensing matrix demonstrated uniform spatial pressure mapping under strain. Integration of the stretchable piezoelectric sensor into a soft gripper further demonstrated real‐time sensing during grasping and release. This work establishes a scalable and automatable manufacturing platform that bridges structural compliance, electronic functionality, and system‐level integration for soft robotics, wearable electronics, and human‐machine interfaces, where stable pressure sensing under large strain is critically needed.

## 9 Materials and Methods

### 9.1 Materials and Electrospinning Solution Preparation

PVdF pellets with a molecular weight (M_n_) of 275,000 g mol^−1^ were purchased from Sigma‐Aldrich (USA). Acetone (ACE) and N, N‐dimethylformamide (DMF) were used as solvents. A mixed solvent of DMF and ACE (volume ratio 4:6) was prepared, and PVdF pellets were dissolved to obtain a 15 wt% PVdF solution. The PVdF solution was magnetically stirred at 67°C for 3 h until a homogeneous and transparent solution was achieved.

Transparent 60 A TPU filament (CoexFlex) was then dissolved in the PVdF solution to prepare precursor solutions for electrospinning PVdF/TPU composite microfibers. PVdF and TPU were weighed to maintain a total polymer mass of 1500 mg in each formulation. Six PVdF/TPU weight ratios of 1500:0, 1350:150, 1200:300, 1050:450, 900:600, and 750:750 w/w were prepared, corresponding to TPU contents of 0, 10, 20, 30, 40, and 50 wt%, respectively. Each PVdF/TPU solution was prepared at a 15 wt% polymer concentration and magnetically stirred at 67°C for 3 h to obtain a clear and homogeneous solution. The prepared samples included electrospun pure PVdF microfibers and PVdF/TPU composite microfibers with various TPU concentrations. All chemicals and materials were used as received without further purification.

### 9.2 Apparatus for Electrospinning

A standard electrospinning setup was assembled, consisting of a high‐voltage DC power supply (Gamma High Voltage), a syringe pump (KD Scientific), a spinneret, and a conductive collector (200 mm × 200 mm × 0.5 mm). The PVdF solution was loaded into a 5 mL plastic syringe fitted with a 21‐gauge stainless‐steel needle (inner diameter 0.51 mm, length 38.1 mm). The solution feed rate was precisely controlled by the syringe pump. A positive potential of 15–25 kV was applied between the needle and the grounded collector to generate the electrospinning jet, and the needle‐to‐collector distance was fixed at 150 mm.

A custom‐designed gap electrospinning system was used to fabricate aligned PVdF and PVdF/TPU composite microfibers. The microfibers were deposited between two parallel metal blades that served as collectors. Positive voltages of 15–25 kV were applied to the needle (Gamma High Voltage), and negative potentials between −1 and −3 kV were applied to the collectors (Trek Model 610E, Trek Inc.). The collector edge radius was 0.25 mm. Voltages lower than ‐4 kV were avoided because air ionization near the blade edges produced repulsive electrostatic forces that disrupted fiber deposition.

The collector gap was adjusted from 10 mm to 100 mm to control fiber span. The working distance between the needle tip and the midpoint of the electrode gap was fixed at 200 mm. The solution feed rate was set to 0.51 mL h^−1^. The applied voltages at the needle tip (V^+^) and the collector plates (V^−^) were adjusted accordingly. All experiments were conducted under ambient conditions with a relative humidity of 55%–60%, as monitored by a digital hygrometer (ThermoPro TP50).

### 9.3 Characterization of Mechanical Properties

A comparative study was conducted to investigate the influence of TPU concentration on the mechanical properties and piezoelectric output of electrospun PVdF/TPU composite microfibers. Uniaxial tensile tests were performed using an ADMET eXpert 2600 dual‐column electromechanical testing system. Each specimen was clamped with a grip separation of 10 mm and tested at a crosshead speed of 12 mm min^−1^ under ambient conditions (25°C). Six specimens from each group were tested to ensure statistical significance. The thickness of each sample, electrospun from PVdF (M_n_ = 275,000 g mol^−1^), was measured using a precision micrometer prior to testing.

### 9.4 Modified 3D Printer for Printing Flexible Filaments

In this work, a commercially available X60 Ultra Flexible Filament (Shore hardness 60 A) was used to print flexible and stretchable 3D architectures. The flexible 3D‐printable filaments were composed of thermoplastic elastomers (TPEs) blended with soft rubber materials. Incorporating soft rubber components adjusted the mechanical properties, such as Young's modulus, of the composite filament, thereby improving stretchability. A LulzBot TAZ 6 3D printer was modified for *m*‐MEX using a commercially available X60 Ultra Flexible TPU filament (shown in Figure S11). A Flexion Extruder designed for flexible materials was installed to enable smooth extrusion and high‐quality printing. The extruder employed a high‐stiffness lever and a cam‐screw compression mechanism to precisely control filament feeding and nozzle pressure, which prevented slippage common in conventional spring‐loaded extruders. All flexible 3D architectures in this study were printed using the modified LulzBot TAZ 6 printer with the following parameters: infill density 90%, layer height 0.15 mm, build‐plate temperature 60°C, nozzle temperature 205°C, and print speed 10 mm s^−1^.

### 9.5 Fully Additively Manufactured Stretchable Piezoelectric Pressure Sensors

The fabrication process consisted of three main steps. (1) A 15 wt% PVdF/TPU solution (PVdF 750 mg, TPU 750 mg) in a 10 mL DMF/ACE (4:6 v/v) solvent mixture was prepared and electrospun via gap electrospinning to form aligned PVdF/TPU microfiber membranes. (2) *l*‐MEX printing was applied to print stretchable silver‐paste electrodes on both sides of the membrane, forming the core piezoelectric sensing layer. Electrical wiring was then integrated to connect the PVdF/TPU sensor to the signal‐conditioning circuit. The stretchable axis was oriented parallel to the applied load and perpendicular to the microfiber alignment direction, corresponding to the direction of the lowest Young's modulus. (3) Soft TPU 3D architectures were printed using the modified *m*‐MEX printer and integrated with the electrospun PVdF/TPU sensor to form the complete stretchable piezoelectric pressure sensor. The assembled structure was encapsulated with polydimethylsiloxane (PDMS) (SYLGARD 184 Silicone Elastomer Kit, Dow Corning) to provide environmental protection and mechanical durability. The PDMS encapsulation layer was used to provide mechanical protection, electrical insulation, and structural integration of the printed sensor layers; however, the sensor performance reported in this work was evaluated under controlled laboratory conditions, with the relative humidity maintained between 55.0% and 60.0% as measured by a digital humidity gauge (ThermoPro TP50). Systematic characterization of temperature‐ and humidity‐induced baseline drift and sensitivity variation will be required for practical deployment in complex environments.

### 9.6 Characterization of Piezoelectric Output During Stretching

The stretch‐induced strain‐insensitive performance of the stretchable sensor under the piezoelectric sensing mode was characterized using a custom‐built testing setup equipped with a pressure‐application module and a charge‐amplifier circuit. Pressures (applied at a frequency of 0.5 Hz) were cyclically applied to the sensing area, with each cycle consisting of 1 s loading followed by 1 s unloading. The applied pressure generated two voltage spikes: a positive spike during loading and a negative spike during unloading, consistent with the typical response of piezoelectric materials. During testing, the stretchable sensor was mounted on a linear stretcher to impose controlled uniaxial stretching. Known pressures were sequentially applied at strains of 0%, 10%, 20%, and 30%. Calibration curves were obtained by plotting peak output voltage as a function of applied pressure under each strain condition, enabling direct comparison of strain‐insensitive performance.

### 9.7 Characterization of Capacitance During Stretching

The stretch‐induced strain‐insensitive performance of the stretchable sensor under the capacitive sensing mode was evaluated using an LCR meter (NF ZM 2372) over a frequency range of 1–10^5^ Hz at 25°C. The stretchable pressure sensor was mounted on the same linear stretcher, and known pressures were applied by placing calibrated weights onto the sensing area. The corresponding capacitances were recorded at strains of 0%, 10%, 20%, and 30%. Calibration curves, plotted as capacitance (measured at 10^3^ Hz) versus pressure, were compared to assess stability and strain insensitivity under mechanical deformation.

### 9.8 Proof‐of‐Concept Demonstration

The control system (Figure 4e), constructed to manipulate the soft gripper and acquire corresponding piezoelectric signals during operation, consisted of an electrical signal acquisition section and a mechanical actuation section. The electrical section included (S1) flexible and stretchable PVdF pressure‐sensing devices, (S2) charge‐amplifier circuits, (S3) a National Instruments USB‐6210 multifunction data acquisition (DAQ) device, and (S4) a laptop computer used for Arduino programming and signal acquisition via LabVIEW 2017 software. The mechanical section comprised (M1) a sample holder, (M2) the soft gripper, (M3) a fixed pulley, (M4) a Dynamixel AX‐12A robotic actuator, (M5) an adjustable DC power supply, and (M6) an Arduino Mega 2560 microcontroller operated through the Arduino IDE.

To achieve consistent pressure measurement results during the grasping task, a fixed pneumatic pressure was supplied to drive deformation and grasping of the soft gripper. The height of each object was adjusted prior to grasping to establish line contact between the soft gripper and the object. The height of the gripper was adjusted to match the object level, and pneumatic pressure was applied to drive the soft gripper toward the intended contact line. The pressure subsequently increased to the predefined maximum set point, and the gripper was lifted to perform the grasping task. The piezoelectric output was measured and recorded.

## Author Contributions

**Jinsheng Fan**: conceptualization, methodology, data curation, investigation, validation, formal analysis, writing – original draft. **Shujia Xu**: data curation, formal analysis. **Yi Yang**: investigation, formal analysis. **David Harmon**: resources, investigation. **Yuelin Deng**: methodology, data curation, investigation. **Brittany Newell**: resources, methodology, supervision. **Wenzhuo Wu**: resources, supervision. **Robert A. Nawrocki**: resources, conceptualization, methodology, supervision, formal analysis, project administration, writing – original draft.

## Funding

This work was supported, in part, by the National Science Foundation (NSF) under Grant CNS‐1726865 and by the NSF Center for Robots and Sensors for the Human Well‐Being (RoSe‐HUB) under Grant CNS‐1439717. Additional support was provided by the 2020 SEED Grant from the Purdue Polytechnic Institute's Realizing the Digital Enterprise initiative. Support for Dr. J. Fan was provided by the Bilsland Dissertation Fellowship at Purdue University.

## Supporting information




**Supporting File 1**: smll74747‐sup‐0001‐SuppMat.pdf.

**Supporting File 2**: smll74747‐sup‐0002‐MovieS1.MOV.

## Data Availability

The data that support the findings of this study are available from the corresponding author upon reasonable request.
