# Supplementary material for: Material Extrusion‐Enabled Soft Multimodal Pressure Sensors With Fabrication‐Embedded Stretch‐Induced Strain Insensitivity
Source: Small. 2026 Jul 21;22(52):e74747. doi: 10.1002/smll.74747 (PMC13580286; doi:10.1002/smll.74747)
Supplement: Supplementary file 1 [file SMLL-22-e74747-s001.pdf]

Supporting Information

Material Extrusion-Enabled Soft Multimodal Pressure Sensors with Fabrication-Embedded Stretch-Induced Strain Insensitivity

*Jinsheng Fan, Shujia Xu, Yi Yang, David Harmon, Yuelin Deng, Brittany Newell, Wenzhuo Wu, and Robert A. Nawrocki**

Jinsheng Fan, Yi Yang, Yuelin Deng, Brittany Newell, Robert A. Nawrocki
School of Engineering Technology, Purdue University, West Lafayette, IN 47907, USA
E-mail: robertnawrocki@purdue.edu (R. A. Nawrocki).

Shujia Xu, Wenzhuo Wu
School of Industrial Engineering, Purdue University, West Lafayette, IN 47907, USA

David Harmon
College of Engineering, Purdue University, West Lafayette, IN 47907, USA

Section S1. Comprehensive Characterization of Stretchable Silver Electrodes

The surface metallization of the electrospun piezoelectric microfiber layer was carried out through *l*-MEX printing of a stretchable conductive silver paste. The experimental plan for testing the performance of the stretchable silver paste is listed in **Table S1**. The geometric dimensions of a stretchable silver electrode test sample are shown in **Fig. S1**. The variation in the conductivity of *l*-MEX printed stretchable electrodes during stretching is plotted in **Fig. S2**. The solid lines represent the mean resistance of each group, with the lower and upper boundaries corresponding to the minimum and maximum values, respectively. Samples within the same group were printed using identical parameters on the same substrate, that is, the same infill direction, and were subjected to the same annealing treatment.

In general, the resistance of the printed stretchable electrodes remained close to the initial resistance at the beginning of stretching. When the deformation ratio ($\Delta L/L_0$) was below 30%, the resistance remained under 100 Ω across all groups. For electrodes printed with Dycotec paste and annealed at 120 °C (Fig. S2a), resistance response differed depending on substrate infill direction (0°, 45°, and 90°). Each group consisted of six samples printed on *m*-MEX printed TPU substrates and annealed at 120 °C for 10 min. Even under identical conditions, resistance values showed considerable variation, which was later quantified by standard deviation. For example, before stretching, the mean resistance of the 90° infill group was 9.99 Ω. At a 30% stretching ratio, one sample measured 22.57 Ω, while another sample in the same group reached 84.05 Ω. When stretched to 60%, the first sample measured 84.91 Ω, whereas the second reached 997.61 Ω.

The standard deviation reflected the variation among the six samples at each stretching ratio. The standard deviation increased exponentially with the stretching ratio. Because this study focused on applications in soft grippers and soft robotics, electrode performance was emphasized in the 0–30% strain range. At 30% strain, the standard deviations of resistance for electrodes printed on 0°, 45°, and 90° infill substrates and annealed at 120 °C were 43.80, 22.32, and 22.18, respectively. These critical values, including the mean, maximum, minimum, and standard deviation, for the 120 °C groups are summarized in **Tables S2, S3,** and **S4**. With higher annealing temperatures, the standard deviations decreased. At 140 °C, the standard deviations fell to 33.14, 10.41, and 9.72 for 0°, 45°, and 90° infill substrates, respectively (Fig. S2b; **Tables S5, S6,** and **S7**). At 160 °C, the standard deviations at 30% strain were further reduced to 9.86, 4.30, and 2.33 for 0°, 45°, and 90° infill substrates, respectively (Fig. S2c; **Tables S8, S9,** and

S10). The decrease in standard deviation with annealing temperature indicated that electrode performance became more consistent across samples prepared under the same conditions.

Besides standard deviation, the change in resistance (ΔR) during stretching was also important because it directly reflected electrode performance. ΔR was calculated as the resistance at a given strain minus the initial resistance value. In general, ΔR increased with the stretching ratio. For samples annealed at the same temperature, resistance stability differed among substrates with different infill directions. Electrodes printed on 90° infill substrates exhibited relatively stable resistance compared with those printed on 0° and 45° infill substrates, indicating that the corn-row-like structure of the printed substrate strongly influenced electrode performance during stretching.

For electrodes printed on substrates with the same infill direction, a higher annealing temperature improved resistance stability. For example, at 30% strain, the percentage resistance change ($\Delta R/R_0$) for electrodes printed on 90° infill substrates was 364.49% at 120 °C, decreasing to 273.32% at 140 °C and 218.47% at 160 °C. A similar trend was observed for the 0° and 45° groups. Overall, electrodes printed on 90° infill substrates and annealed at 160 °C exhibited the best performance, defined as the lowest rate of resistance increase with strain.

For comparison, the performance of a DuPont silver paste was also studied. Electrodes were printed only on 90° infill substrates and annealed at 130 °C for 15 min, following the manufacturer's datasheet specifications. The result is shown in Fig. S2d. The performance values are summarized in **Table S11**. Compared with Dycotec ink, electrodes printed with DuPont ink showed superior stability. As strain increased from 0% to 50%, the $\Delta R/R_0$ ratio rose from 0% to 674.41%, which was about half that of the best-performing Dycotec sample (1271.92%).

The printed Ag electrode was prepared using a commercial stretchable conductive ink and was used as a functional stretchable electrode in this work. The resistance measurements are used to verify electrode functionality and stretchability within the deformation range of this study, rather than to establish the intrinsic deformation mechanism of the commercial Ag ink. The observed resistance increase under tensile strain may result from conductive-pathway elongation, localized microcrack formation, and/or rearrangement of the Ag percolation network. Because the ink formulation, filler morphology, binder composition, and conductive-network structure are unknown, the microscopic origin of the resistance change cannot be conclusively determined.

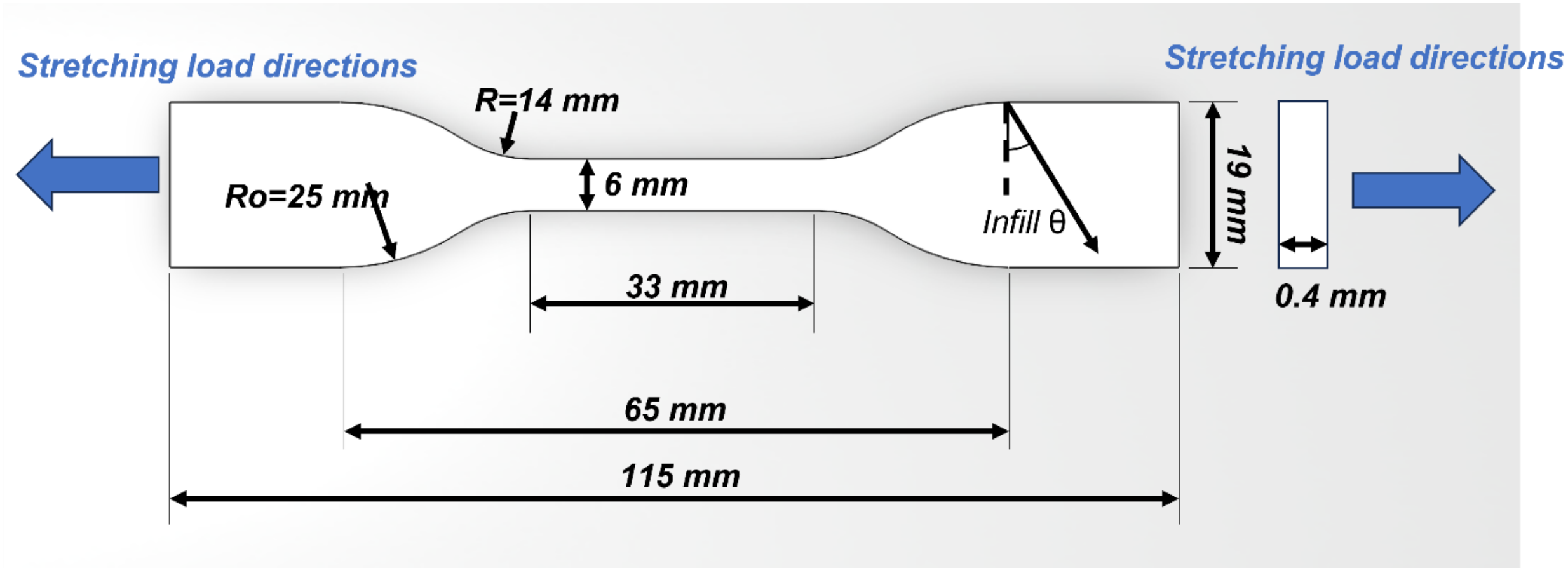

FIGURE S1. Dimensions of *m*-MEX printed TPU substrate and the definition of infill directions.

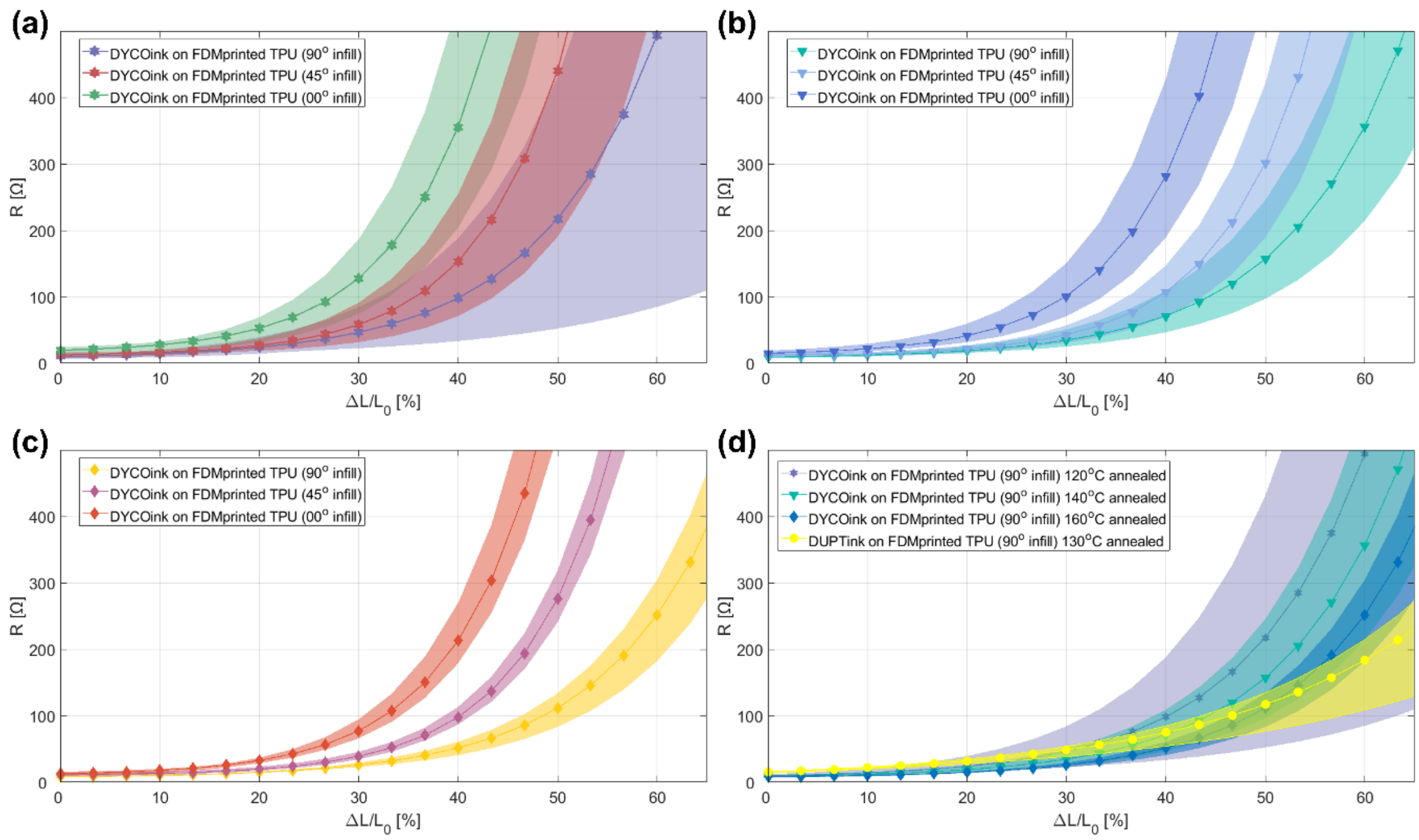

FIGURE S2. Variations in resistance of *l*-MEX printed silver electrodes as a function of stretching ratios. (a) Dycotec silver electrodes printed on TPU substrates with different infill directions and annealed at 120 °C, (b) annealed at 140 °C, (c) annealed at 160 °C, and (d) comparison between the performance of Dycotec and DuPont silver paste electrodes printed on TPU with the same infill direction.

TABLE S1. Experimental plan for testing the performance of stretchable silver paste.

Group #	Ink manufacturer	Substrate infill [°]	Annealing temperature [°C]	Time [min]
1	Dycotec	0	120	10
2	Dycotec	45	120	10
3	Dycotec	90	120	10
4	Dycotec	0	140	10
5	Dycotec	45	140	10
6	Dycotec	90	140	10
7	Dycotec	0	160	10
8	Dycotec	45	160	10
9	Dycotec	90	160	10
10	DuPont	90	130	15

TABLE S2. Performance of Dycotec electrodes on 90° infill substrates annealed at 120 °C.

$\Delta L/L_0$ [%]	Mean [Ω]	Minimum [Ω]	Maximum [Ω]	SD*	ΔR [Ω]	$\Delta R/R_0$ [%]
0	9.99	6.70	12.10	1.96	0.00	0.00
10	14.27	9.03	20.30	3.84	4.27	42.74
20	24.02	14.48	39.43	9.07	14.03	140.37
30	46.42	22.57	84.05	22.18	36.43	364.49
40	98.04	33.44	188.16	53.93	88.05	881.04
50	217.39	52.27	431.03	129.86	207.39	2075.19
60	493.91	84.91	997.61	310.15	483.92	4842.09

*SD is the abbreviation of standard deviation.

TABLE S3. Performance of Dycotec electrodes on 45° infill substrates annealed at 120 °C.

$\Delta L/L_0$ [%]	Mean [Ω]	Minimum [Ω]	Maximum [Ω]	SD	ΔR [Ω]	$\Delta R/R_0$ [%]
0	12.95	7.33	16.33	3.05	0.00	0.00
10	16.36	11.18	19.39	3.20	3.41	26.34
20	26.69	18.82	37.04	7.01	13.75	106.20
30	58.04	31.80	91.11	22.32	45.09	348.30
40	153.06	71.15	255.02	70.01	140.11	1082.32
50	441.14	190.43	751.96	214.99	428.20	3307.69

60	1314.56	552.09	2258.57	654.63	1301.62	10054.53

TABLE S4. Performance of Dycotec electrodes on 0° infill substrates annealed at 120 °C.

$\Delta L/L_0$ [%]	Mean [Ω]	Minimum [Ω]	Maximum [Ω]	SD	ΔR [Ω]	$\Delta R/R_0$ [%]
0	19.46	12.73	24.33	3.98	0.00	0.00
10	27.65	17.39	32.40	5.71	8.18	42.05
20	52.46	31.53	69.29	14.52	33.00	169.55
30	127.69	74.39	186.86	43.80	108.23	556.09
40	355.77	204.32	543.27	133.55	336.31	1728.00
50	1047.26	598.27	1623.86	405.94	1027.80	5280.97
60	3143.70	1792.62	4899.95	1231.87	3124.24	16052.81

TABLE S5. Performance of Dycotec electrodes on 90° infill substrates annealed at 140 °C.

$\Delta L/L_0$ [%]	Mean [Ω]	Minimum [Ω]	Maximum [Ω]	SD	ΔR [Ω]	$\Delta R/R_0$ [%]
0	9.28	7.75	10.40	1.13	0.00	0.00
10	12.17	10.15	15.00	1.77	2.89	31.15
20	18.91	15.52	25.73	4.03	9.63	103.81
30	34.64	25.13	50.78	9.72	25.36	273.32
40	71.33	46.71	109.20	23.16	62.05	668.77
50	156.93	97.05	245.48	54.60	147.65	1591.28
60	356.62	214.49	563.42	127.96	347.34	3743.36

TABLE S6. Performance of Dycotec electrodes on 45° infill substrates annealed at 140 °C.

$\Delta L/L_0$ [%]	Mean [Ω]	Minimum [Ω]	Maximum [Ω]	SD	ΔR [Ω]	$\Delta R/R_0$ [%]
0	12.40	9.26	14.03	1.81	0.00	0.00
10	14.70	10.95	17.27	2.34	2.30	18.57
20	21.68	16.06	27.09	4.22	9.28	74.85
30	42.84	30.80	56.88	10.41	30.44	245.50
40	107.00	70.15	147.18	29.50	94.60	762.88
50	301.51	189.43	420.97	87.53	289.11	2331.44
60	891.22	551.09	1251.01	263.51	878.82	7086.98

TABLE S7. Performance of Dycotec electrodes on 0° infill substrates annealed at 140 °C.

$\Delta L/L_0$ [%]	Mean [Ω]	Minimum [Ω]	Maximum [Ω]	SD	ΔR [Ω]	$\Delta R/R_0$ [%]
0	15.13	10.73	19.33	2.77	0.00	0.00
10	21.62	17.42	29.27	4.46	6.49	42.91
20	41.30	31.69	59.43	11.28	26.17	172.99
30	100.97	70.81	150.84	33.14	85.84	567.38
40	281.86	189.43	427.99	99.88	266.73	1763.08
50	830.30	549.04	1268.25	302.39	815.17	5388.18
60	2493.05	1639.31	3815.74	916.41	2477.92	16378.70

TABLE S8. Performance of Dycotec electrodes on 90° infill substrates annealed at 160 °C.

$\Delta L/L_0$ [%]	Mean [Ω]	Minimum [Ω]	Maximum [Ω]	SD	ΔR [Ω]	$\Delta R/R_0$ [%]
0	8.13	7.40	10.02	0.99	0.00	0.00
10	10.15	9.33	11.45	0.75	2.02	24.90
20	14.87	13.85	16.03	0.70	6.75	82.98
30	25.89	22.59	29.46	2.33	17.76	218.47
40	51.58	40.79	60.81	6.69	43.45	534.55
50	111.52	83.23	133.94	16.95	103.39	1271.92
60	251.35	182.25	304.53	40.94	243.22	2992.10

TABLE S9. Performance of Dycotec electrodes on 45° infill substrates annealed at 160 °C.

$\Delta L/L_0$ [%]	Mean [Ω]	Minimum [Ω]	Maximum [Ω]	SD	ΔR [Ω]	$\Delta R/R_0$ [%]
0	11.14	9.36	13.63	1.55	0.00	0.00
10	13.25	11.25	16.06	1.67	2.11	18.94
20	19.64	16.98	23.42	2.18	8.50	76.34
30	39.03	34.34	45.73	4.30	27.89	250.40
40	97.81	86.96	113.39	11.46	86.67	778.08
50	276.01	240.66	318.51	33.57	264.87	2377.91
60	816.27	706.41	940.38	100.76	805.14	7228.23

TABLE S10. Performance of Dycotec electrodes on 0° infill substrates annealed at 160 °C.

$\Delta L/L_0$ [%]	Mean [Ω]	Minimum [Ω]	Maximum [Ω]	SD	ΔR [Ω]	$\Delta R/R_0$ [%]
0	13.00	9.73	15.63	2.19	0.00	0.00

10	17.86	14.39	20.09	2.12	4.87	37.44
20	32.61	28.53	36.88	3.30	19.62	150.96
30	77.34	66.03	94.68	9.86	64.34	495.13
40	212.94	178.98	269.91	31.18	199.94	1538.56
50	624.05	521.42	801.19	96.28	611.06	4702.02
60	1870.45	1559.65	2411.89	293.75	1857.45	14292.95

TABLE S11. Performance of DuPont electrodes on 90° infill substrates annealed at 130 °C.

$\Delta L/L_0$ [%]	Mean [Ω]	Minimum [Ω]	Maximum [Ω]	SD	ΔR [Ω]	$\Delta R/R_0$ [%]
0	15.08	13.79	15.93	0.88	0.00	0.00
10	21.63	19.79	22.93	1.29	6.55	43.46
20	32.03	27.49	34.80	2.59	16.95	112.42
30	48.56	38.48	53.94	5.42	33.48	222.01
40	74.86	54.18	84.78	10.83	59.78	396.44
50	116.78	76.61	134.51	20.71	101.70	674.41
60	183.66	108.65	214.66	38.28	168.58	1117.93

Section S2. Signal-Conditioning Circuit for Piezoelectric Pressure Sensors

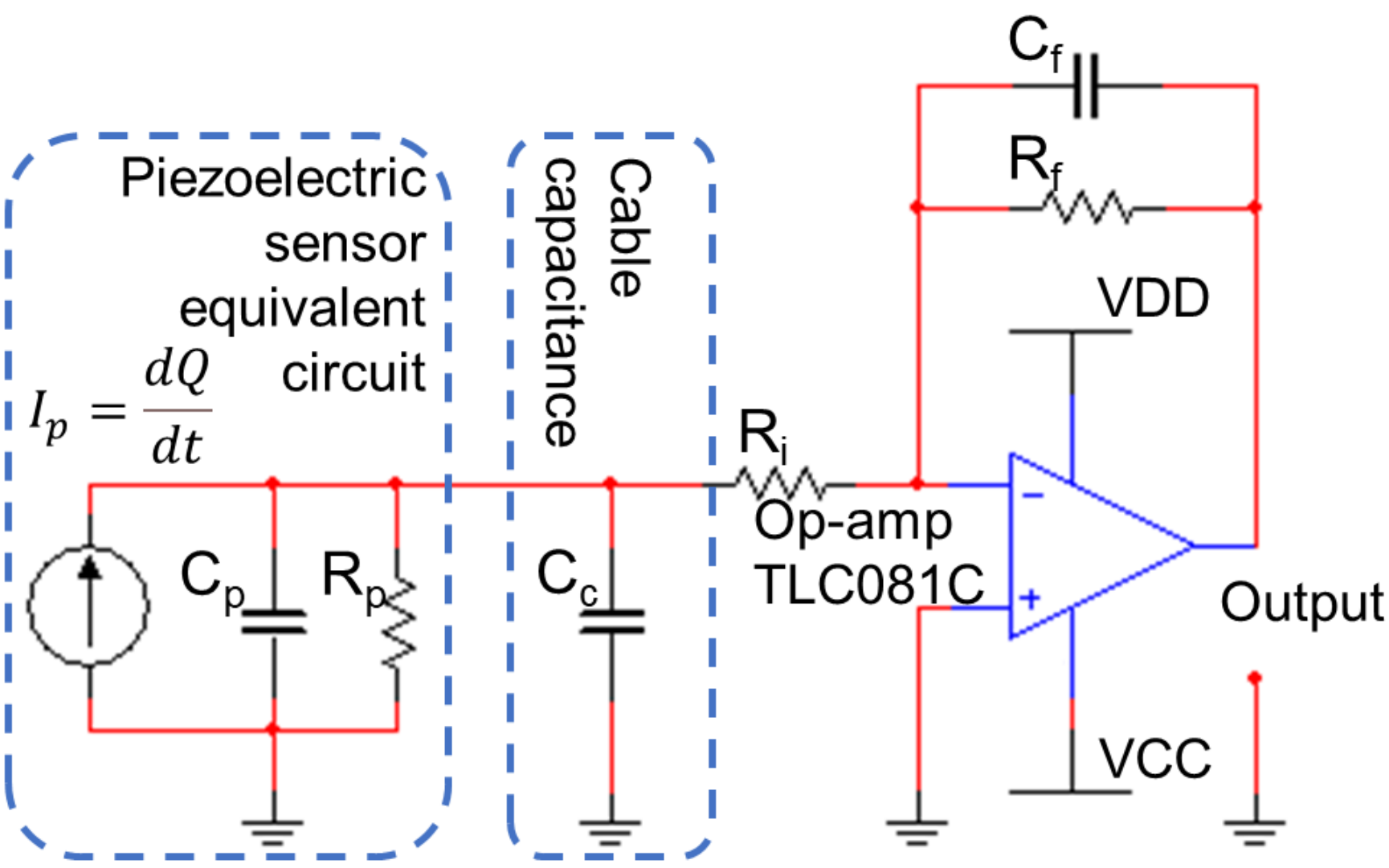

FIGURE S3. Charge-amplifier circuit for piezoelectric output voltage measurement.

Section S3. Supplementary Validation of Stretch-Induced Strain-Insensitive Pressure Sensing

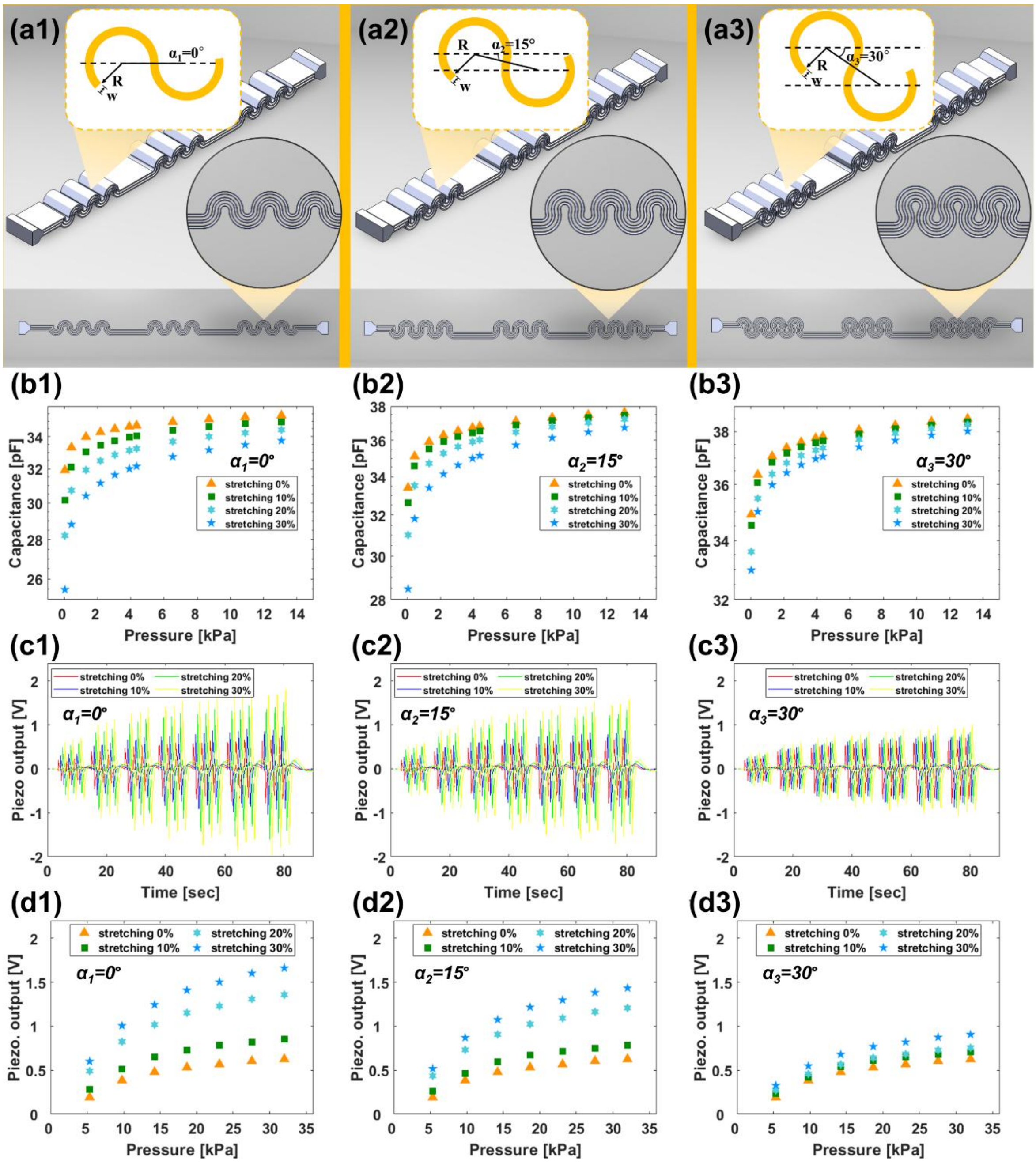

FIGURE S4. Validation of stretch-induced strain-insensitive pressure sensing enabled by arc-opening-angle engineering of the *m*-MEX printed serpentine architecture. (a) CAD models of serpentine architectures with arc opening angles (α) of 0°, 15°, and 30°, illustrating the in-plane geometric parameters, including arc radius (*R*), width (*w*), and arc opening angle (*α*). (b) Capacitance–pressure calibration curves, (c) piezoelectric output voltage recorded under stepwise increasing pressure, and (d) piezoelectric output–pressure calibration curves measured at applied strains of 0%, 10%, 20%, and 30% for sensors with different arc opening angles.

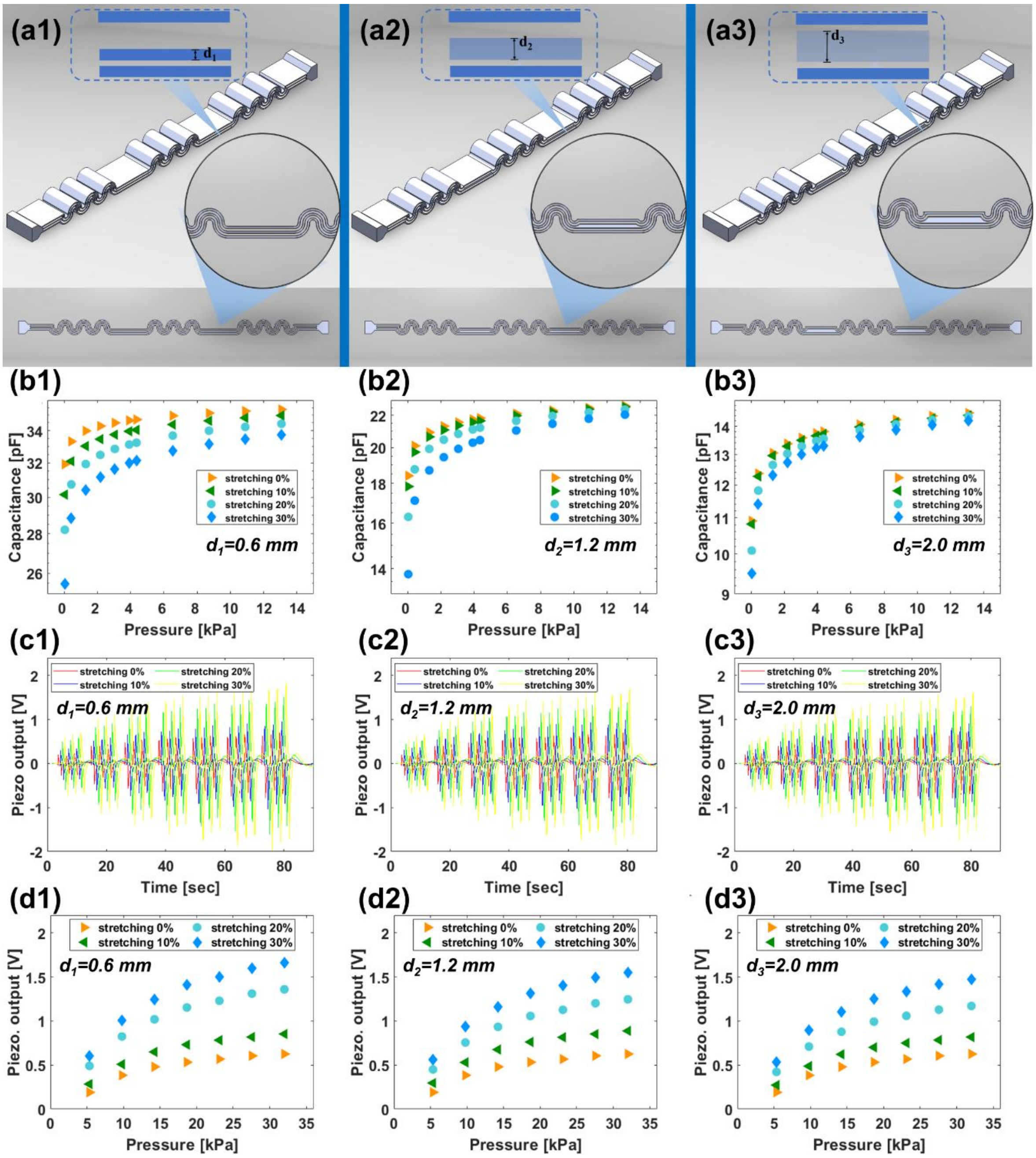

FIGURE S5. Validation of stretch-induced strain-insensitive pressure sensing enabled by center-layer-thickness engineering of the *m*-MEX printed serpentine architecture. (a) CAD models of serpentine architectures with center-layer thicknesses (*d*) of 0.6, 1.2, and 2.0 mm. (b) Capacitance–pressure calibration curves, (c) piezoelectric output voltage recorded under stepwise increasing pressure, and (d) piezoelectric output–pressure calibration curves for sensors with different center-layer thicknesses, measured at applied strains of 0%, 10%, 20%, and 30%.

Section S4. Supplementary Comparison with State-of-the-Art Strain-Insensitive and Stretchable Pressure Sensors

TABLE S12. Comparison with state-of-the-art strain-insensitive and stretchable pressure sensors.

Reference	Sensing mechanism	Manufacturing method	Maximum strain	Strain-insensitive performance	Sensitivity	Detection limit
Su et al.[1]	Iontronic capacitive	Microfabrication and layer-by-layer assembly of ionic dielectric and stretchable electrodes	50%	98% strain insensitivity at 50% strain	4.5 kPa^{-1} (0-1 kPa); 2.0 kPa^{-1} (1-10 kPa)	0.2 Pa
Xia et al.[2]	Piezoresistive	Digital light processing (DLP) 3D printing and conductive-resin infusion	20%	Resistance change of 0.76%; pressure-sensitivity change of 0.22% from 0% to 20% strain	1.15 MPa^{-1} (0-100 kPa)	N/R
Yuan et al.[3]	Piezoresistive	MXene coating, PI micropore isolation, and laser-patterned PI/Cu electrode assembly	45%	Stable pressure sensing under stretching up to 45% and bending curvature up to 256 m^{-1}	21.57 kPa^{-1} (0.5-410 kPa)	140 Pa
Yang et al.[4]	Piezoresistive	CO_2 laser cutting/engraving of Nomex-derived graphene and Ecoflex encapsulation	80%	Relative resistance variation of 0.30% under 80% tensile strain	0.141 kPa^{-1} (0-7 kPa); 0.0132 kPa^{-1} (7-50 kPa)	100 Pa
Ha et al.[5]	Piezoresistive and capacitive	Lamination of CNT-PDMS electrodes, porous nanocomposite, and ultrathin PDMS dielectric layer	70%	Stretch-induced capacitance change from 0% to 40% strain was only 1.8% of the pressure-induced capacitance change from 0 to 50 kPa	1.25 kPa^{-1} (0-10 kPa)	5.22 Pa
Li et al.[6]	Piezoelectric	PEALD-assisted ZnO seed-layer patterning, hydrothermal ZnO nanorod growth, AgNW electrode deposition, and Ecoflex encapsulation	100%	98.2% strain insensitivity under 0-100% strain; output deviation within 7% during dynamic stretching	20.13 V MPa^{-1} (3-40 kPa)	1 Pa
Dong et al.[7]	Capacitive	Acoustic-field assembly of liquid metal composite electrodes and molded pyramid LMC dielectric layer	100%	Consistent capacitance-pressure response from 0% to 100% strain; capacitance-response deviation <5%	0.4 kPa^{-1} (0-10 kPa); 0.2 kPa^{-1} (30-50 kPa)	100 Pa
Guo et al.[8]	Piezoresistive	Magnetic alignment of iron particles, acoustic assembly of liquid-metal network, and laser patterning for arrays	80%	Stable pressure output under tensile strain up to 80%	251.76 MPa^{-1} around 0.45 MPa; 1461.19 MPa^{-1} around 1.51 MPa	400 Pa
**This work**	**Capacitive and piezoelectric**	**Gap electrospinning, liquid material extrusion, and melt material extrusion**	**30%**	**97.6% strain insensitivity under 30% strain in capacitive mode; 85.4% strain-insensitivity ratio at 10% strain in piezoelectric mode**	**Capacitive: 4.9×10^{-2} kPa^{-1} (0-2 kPa), 2.8×10^{-3} kPa^{-1} (2-14 kPa); Piezoelectric: 16.5 mV kPa^{-1}**	**40 Pa**

Note: N/R: not reported. Sensitivity values from different sensing mechanisms use different definitions and units and should not be directly ranked without considering the sensing principle and pressure range. Strain-insensitive performance should be reported as a percentage calculated from the relative change in sensor output under strain. The definition can be written as:

$$\text{Strain} - \text{insensitive performance} = \left(1 - \frac{|S_{\varepsilon_{max}} - S_{0\%}|}{S_{0\%}}\right) \times 100\% \qquad \textbf{Equation S1}$$

where $S_{0\%}$ is the sensor output measured at 0% strain, and $S_{\varepsilon_{max}}$ is the sensor output measured at the maximum applied strain. For Ha et al. and Guo et al., different comparison definitions were used in the original studies; therefore, their reported metrics were retained for accuracy.

Section S5. Supplementary Dynamic Durability Under Cyclic Press-Release and Tensile-Release Deformation

The dynamic durability of the stretchable pressure sensor with an arc opening angle of α_3 = 30° was evaluated through cyclic press-release and tensile-release fatigue testing. First, the sensor was subjected to cyclic press-release loading under a pressure of 60 kPa at both 0% and 30% strain. At 0% strain, the sensor was tested for 14,000 s, corresponding to approximately 233 min. During each cycle, a 4 s waiting period was applied between pressing and release, followed by another 4 s waiting period before the next press-release cycle. Pressing was detected as a positive piezoelectric output peak, while release was detected as a negative piezoelectric output peak. The press-release cycling result at 0% strain is shown in **Fig. S6a**. The average positive and negative peak values were 1.81 V and -0.91 V, respectively, as shown in Fig. S6b.

The same cyclic press-release test was performed while the sensor was held at 30% strain. As shown in **Fig. S7a**, the sensor maintained repeatable press-release responses under the stretched condition. The average positive and negative peak values were 2.42 V and -1.26 V, respectively, as shown in Fig. S7b. The representative response times for pressing and release were 64 ms and 344 ms, respectively, as shown in Fig. S7c.

The sensor was then subjected to 5,000 tensile-release cycles by repeatedly stretching the sensor from 0% to 30% strain and releasing the sensor back to 0% strain at a stretching speed of 10 mm s^{-1}. After tensile-release cycling, the press-release response was measured again under the same 60 kPa cyclic pressure condition. At 0% strain, the post-fatigue response remained stable, as shown in Fig. S6c. The average positive and negative peak values after fatigue testing were 1.72 V and -0.96 V, respectively, as shown in Fig. S6d.

The post-fatigue press-release response was also measured while the sensor was held at 30% strain. As shown in **Fig. S8a**, the sensor still generated clear positive and negative piezoelectric peaks during pressing and release. The corresponding average positive and negative peak values were 2.42 V and -1.25 V, respectively, as shown in Fig. S8b. After tensile-release cycling, the representative response times for pressing and release were 110 ms and 330 ms, respectively, as shown in Fig. S8c.

These results indicated that the sensor retained clear piezoelectric responses to cyclic pressing and release after 5,000 tensile-release cycles. The average output amplitudes and response times remained stable before and after fatigue treatment, suggesting that repeated tensile deformation

did not cause severe irreversible drift or fatigue-induced loss of piezoelectric response under the tested conditions. These data provide additional evidence for the mechanical durability and electromechanical stability of the stretchable pressure sensor during repeated deformation.

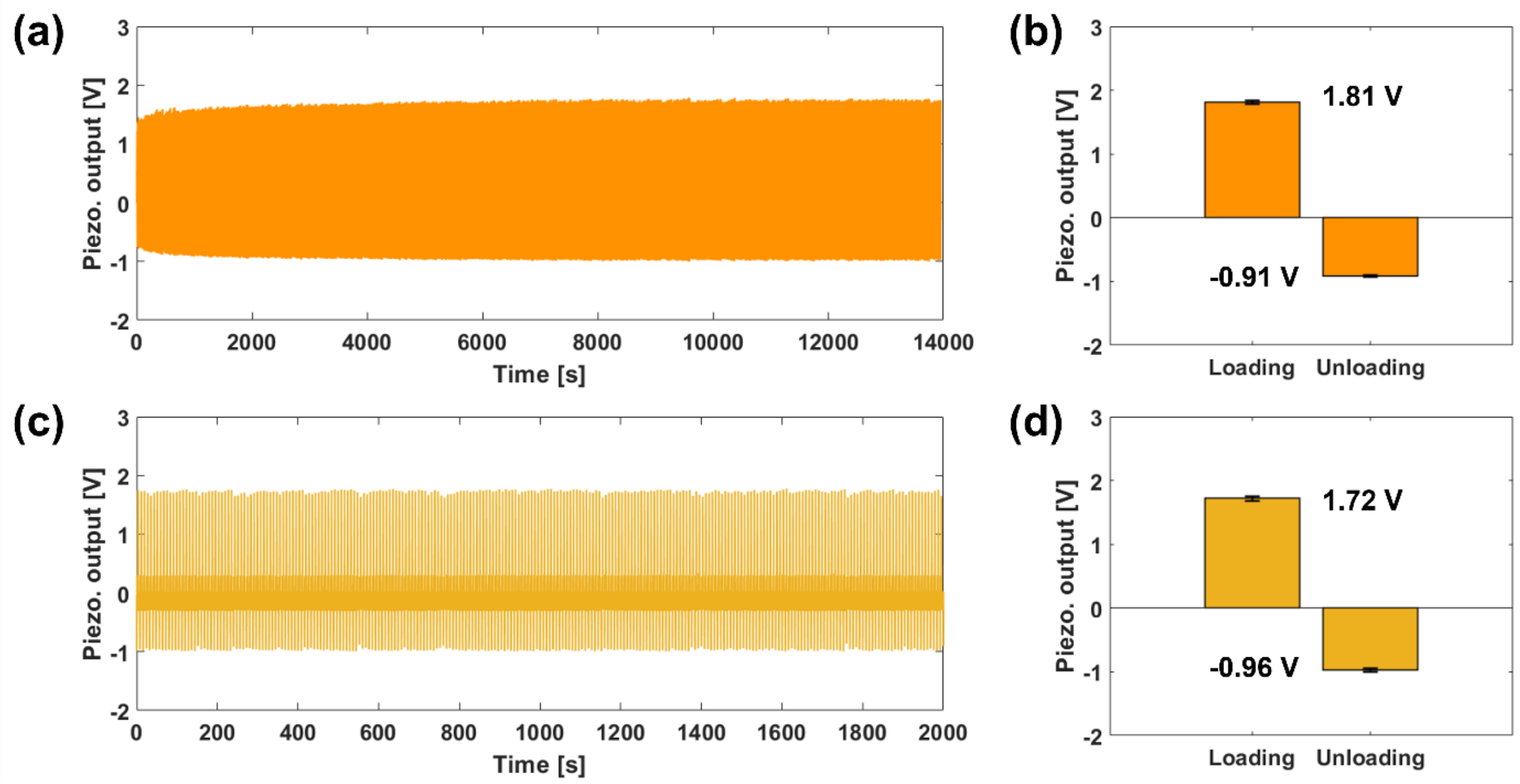

FIGURE S6. Piezoelectric pressure-sensing response of the stretchable sensor before and after 5,000 cyclic tensile-release fatigue cycles. Output signals generated under a cyclic pressure of 60 kPa (a) before and (c) after fatigue treatment. Average output voltage amplitudes during loading and unloading (b) before and (d) after fatigue treatment. (During the 5,000 tensile-release cycles, the sensor was repeatedly stretched from 0% to 30% strain and released back to 0% strain at a stretching speed of 10 mm s^{-1}.)

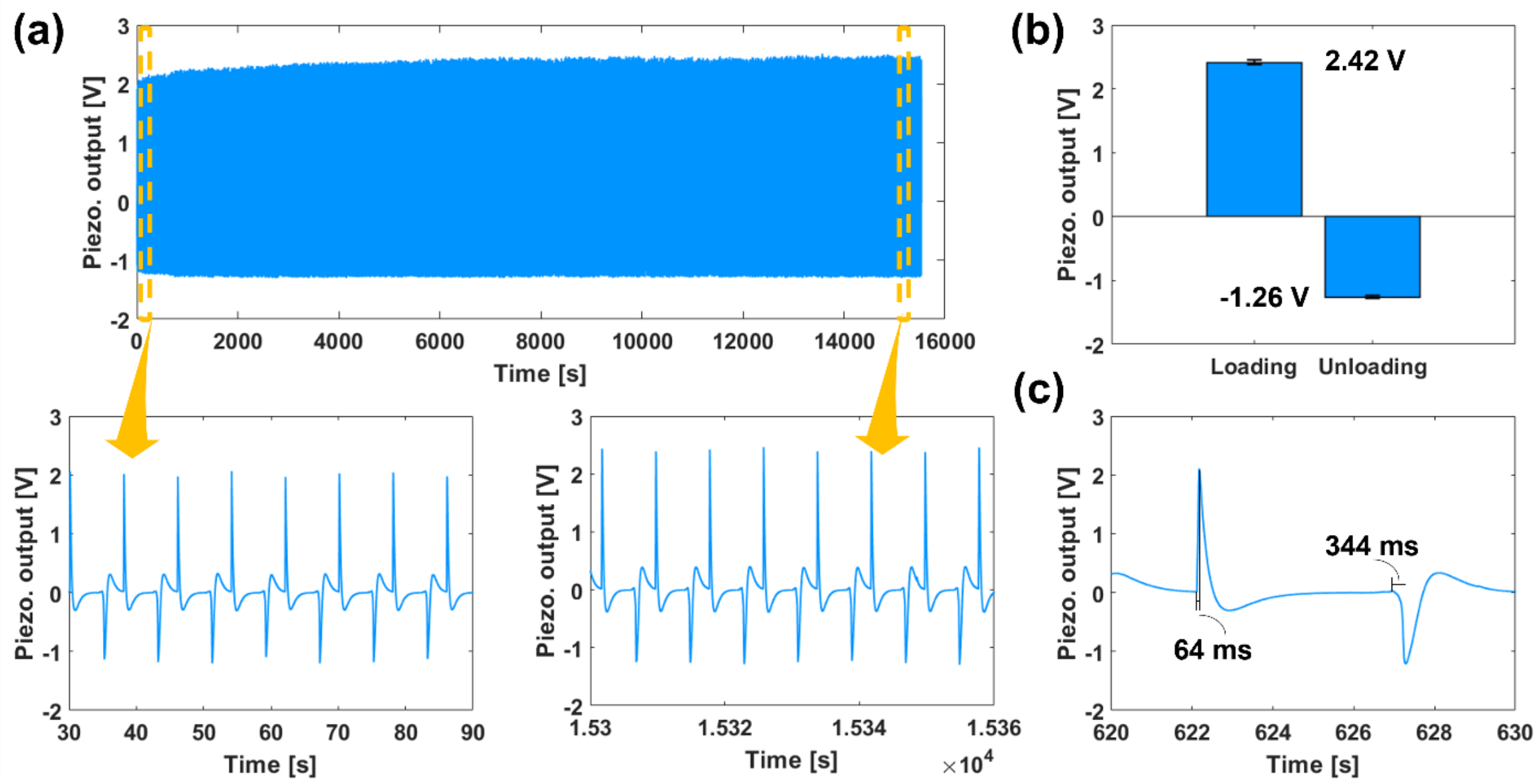

FIGURE S7. Piezoelectric pressure-sensing response of the stretchable sensor under 30% strain before cyclic tensile-release fatigue treatment. (a) Output signals generated under a cyclic

pressure of 60 kPa. (b) Average output voltage amplitudes during loading and unloading. (c) Representative response times of the sensor during loading and unloading.

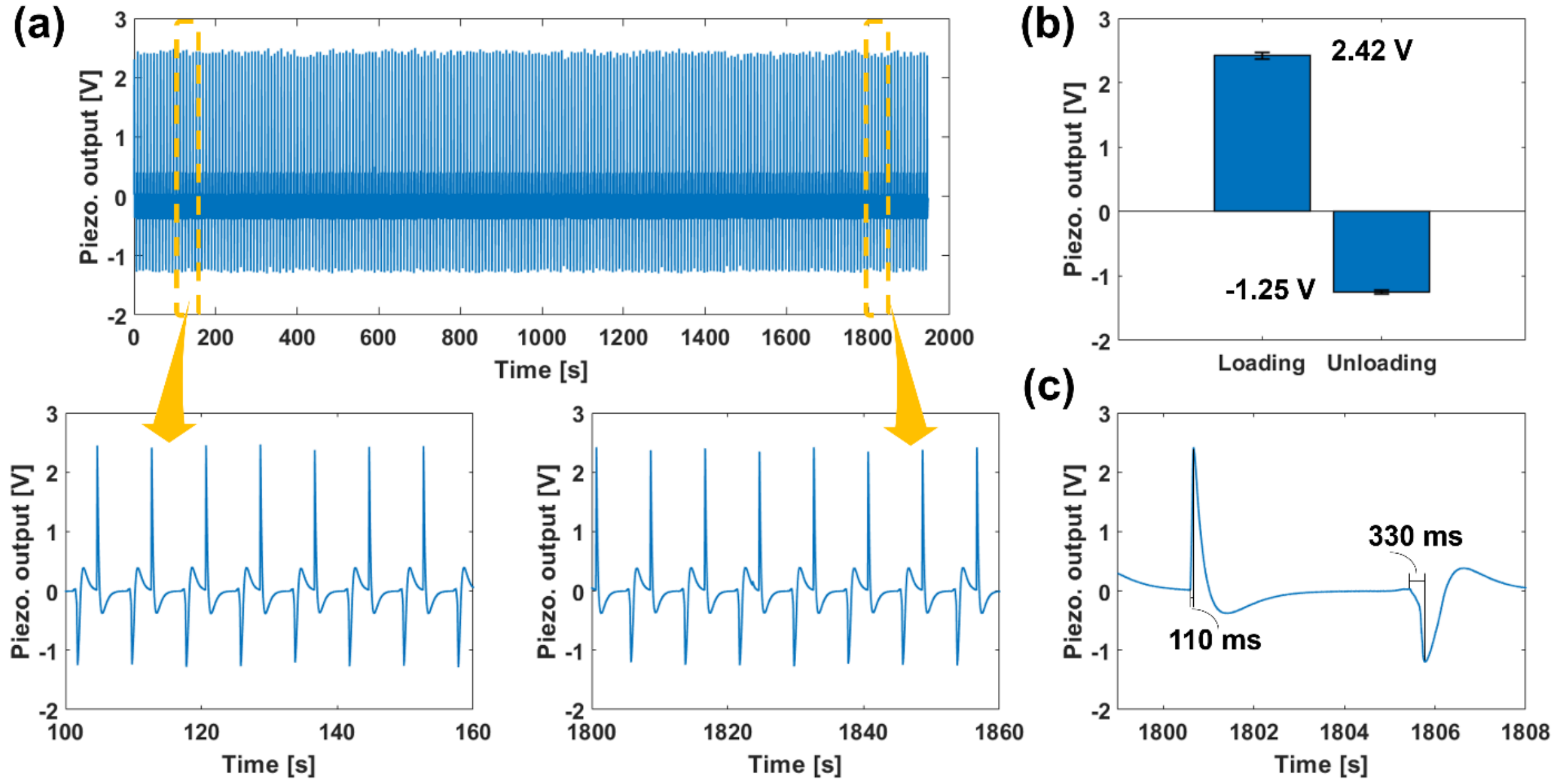

FIGURE S8. Piezoelectric pressure-sensing response of the stretchable sensor under 30% strain after 5,000 tensile-release cycles. (a) Output signals generated under a cyclic pressure of 60 kPa after fatigue treatment. (b) Average output voltage amplitudes during loading and unloading. (c) Representative response times of the sensor during loading and unloading. (During the 5,000 tensile-release cycles, the sensor was repeatedly stretched from 0% to 30% strain and released back to 0% strain at a stretching speed of 10 mm s^{-1}.)

Section S6. Supplementary Fabrication and Characterization of the Stretchable Capacitive Pressure Sensing Matrix

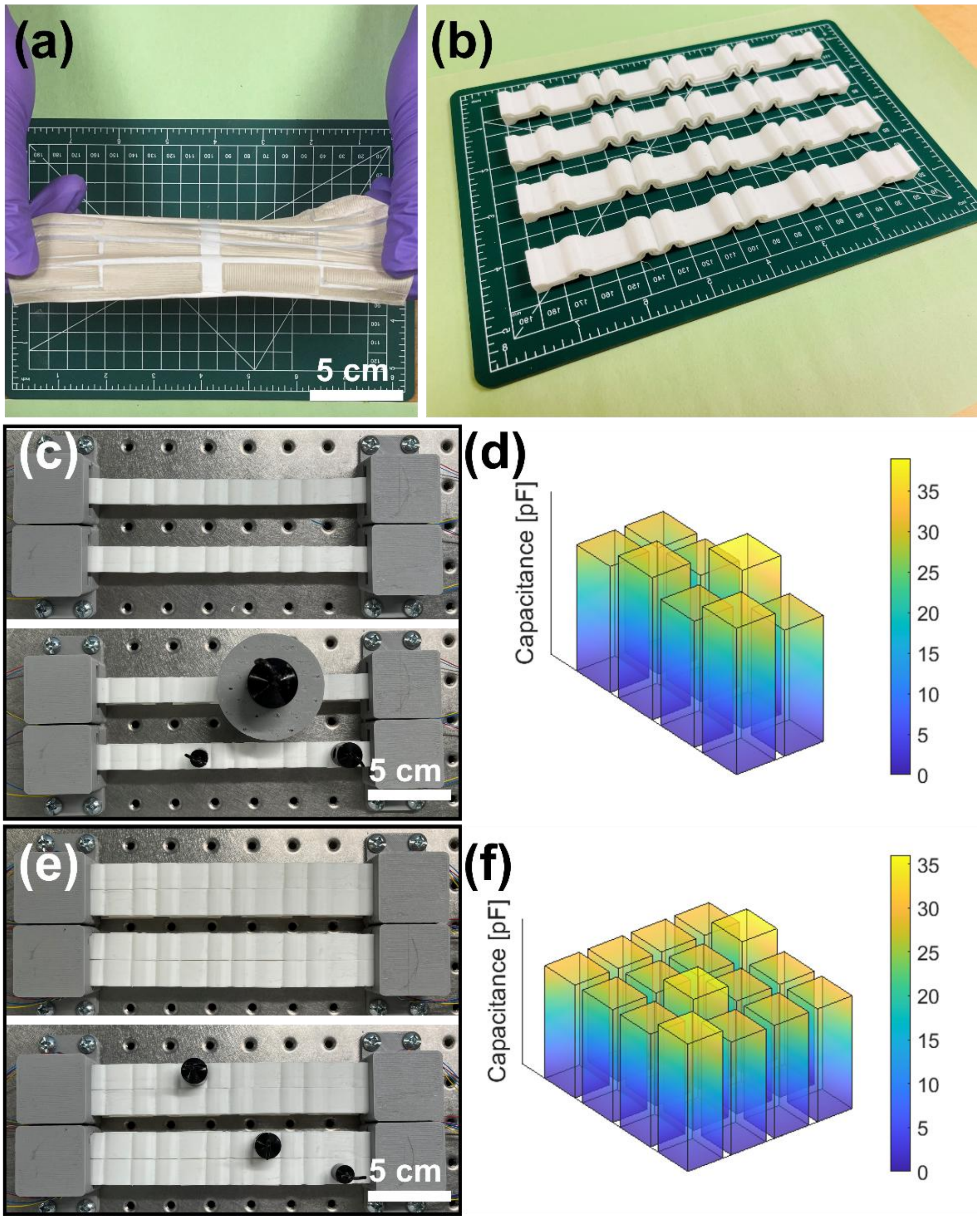

FIGURE S9. Fully additively manufactured electrospun PVdF/TPU composite microfiber-based stretchable capacitive pressure sensing matrix. (a) Electrospun PVdF/TPU composite microfiber membrane with *l*-MEX printed stretchable silver electrode patterns. (b) *m*-MEX printed soft TPU (Shore hardness 60 A) architectures integrating four individual sensors into a single sensing element. (c) Top view of a 2 × 4 capacitive sensing matrix. (d) Capacitance variation of the 2 × 4 matrix under applied static pressures at three sensor nodes. (e) Top view of a 4 × 4 capacitive sensing matrix. (f) Capacitance mapping of the 4 × 4 matrix under applied static pressures at three sensor nodes, confirming the uniformity and spatial selectivity of the sensing array.

Section S7. Supplementary Electromechanical Hysteresis Characterization

The electromechanical hysteresis of the piezoelectric response was evaluated by applying cyclic loading-unloading tests at different speeds and displacements, as shown in **Fig. S10a,c,e**. The corresponding displacement-dependent hysteresis curves were obtained by plotting the piezoelectric output voltage as a function of displacement during cyclic loading and unloading, as shown in Fig. S10b,d,f. The hysteresis error was calculated for each cycle using the maximum voltage difference between the loading and unloading branches at the same displacement, normalized by the full-scale voltage output of the corresponding cycle:

$$H = \frac{max|V_{loading} - V_{unloading}|}{V_{FS}} \times 100\% \qquad \textbf{Equation S2}$$

where $V_{loading}$, and $V_{unloading}$ are the piezoelectric outputs during loading and unloading at the same displacement, respectively, V_{FS} is the full-scale sensor output.

The calculated hysteresis errors are summarized in **Tables S13-S15**. For Fig. S10b, the hysteresis error ranged from 43.31% to 59.71%. For Fig. S10d, the hysteresis error ranged from 50.18% to 60.98%. For Fig. S10f, the hysteresis error ranged from 22.88% to 30.50%. These values quantitatively describe the electromechanical hysteresis of the piezoelectric response during repeated loading and unloading. The measured hysteresis reflects the coupled effects of soft-structure viscoelasticity, dynamic electromechanical coupling of the PVdF/TPU piezoelectric layer, and charge-amplifier-based signal acquisition.

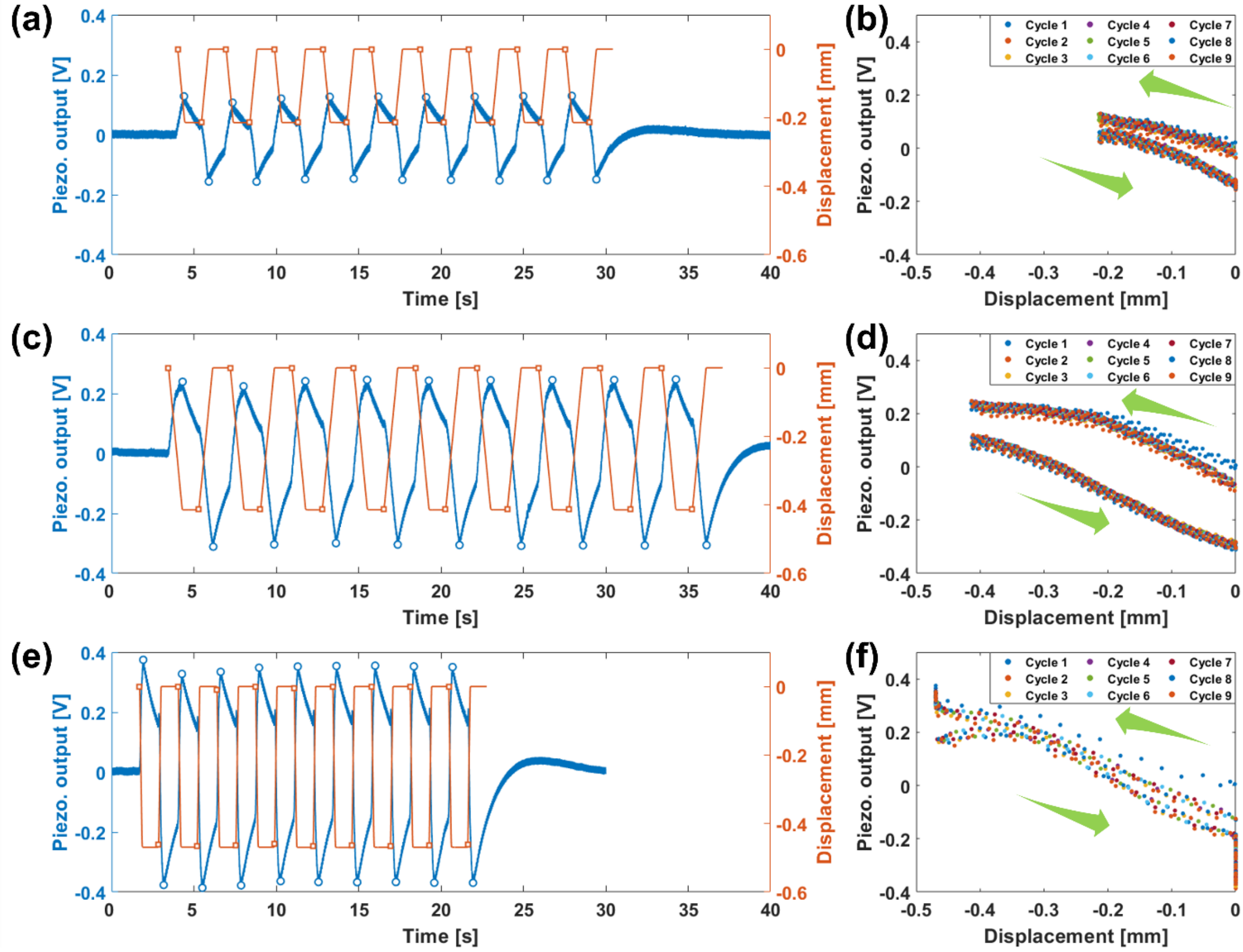

FIGURE S10. Electromechanical hysteresis characterization of the piezoelectric response during cyclic loading and unloading. Time-domain piezoelectric output and displacement responses for three representative tests: (a) average displacement of 0.21 mm, loading speed of 0.47 mm s^{-1}, and unloading speed of 0.43 mm s^{-1}; (c) average displacement of 0.41 mm, loading speed of 0.47 mm s^{-1}, and unloading speed of 0.49 mm s^{-1}; and (e) average displacement of 0.47 mm, loading speed of 2.30 mm s^{-1}, and unloading speed of 2.88 mm s^{-1}. The corresponding piezoelectric output-displacement hysteresis curves for repeated loading-unloading cycles are shown in (b), (d), and (f), respectively.

TABLE S13. Hysteresis error results for Fig. S10b. (Average displacement: 0.21 mm, loading speed: 0.47 mm s^{-1}, and unloading speed: 0.43 mm s^{-1})

Cycle	V_{FS} [V]	Displacement at Max Hysteresis [mm]	Hysteresis Error [%]
1	0.28	-0.02	59.71
2	0.26	-0.00	43.31
3	0.27	-0.01	46.10
4	0.27	-0.02	45.20
5	0.28	-0.00	49.48
6	0.28	-0.01	46.56
7	0.28	-0.02	49.60
8	0.28	-0.00	45.97
9	0.28	-0.02	49.34

TABLE S14. Hysteresis error results for Fig. S10d. (Average displacement: 0.41 mm, loading speed: 0.47 mm s^{-1}, and unloading speed: 0.49 mm s^{-1})

Cycle	V_{FS} [V]	Displacement at Max Hysteresis [mm]	Hysteresis Error [%]
1	0.55	-0.10	60.98
2	0.53	-0.13	52.89
3	0.54	-0.09	51.53
4	0.55	-0.11	52.08
5	0.55	-0.12	53.72
6	0.55	-0.11	50.18
7	0.55	-0.14	54.07
8	0.55	-0.13	51.46
9	0.55	-0.12	53.53

TABLE S15. Hysteresis error results for Fig. S10f. (Average displacement: 0.47 mm, loading speed: 2.30 mm s^{-1}, and unloading speed: 2.88 mm s^{-1})

Cycle	V_{FS} [V]	Displacement at Max Hysteresis [mm]	Hysteresis Error [%]
1	0.75	-0.47	30.50
2	0.71	-0.00	28.12
3	0.71	-0.47	23.41
4	0.71	-0.00	26.26
5	0.72	-0.47	26.46
6	0.72	-0.47	26.91
7	0.72	-0.47	23.52
8	0.72	-0.47	22.88
9	0.72	-0.00	24.57

Section S8. Analytical Derivation of the Effective Stiffness of Stacked Freestanding Serpentine Ribbons

A unit cell cut out of a one-directional periodic serpentine ribbon is composed of an arc joined end-to-end with its upside-down mirror image. The unit cell of this serpentine can be well defined by four geometric parameters: the ribbon width w, the arc radius R, the arc angle α, and the arm length l. The ribbon thickness t is assumed to be unity in the plane strain model. When this unit cell is subjected to a tensile displacement δ_0 at each end, the effective applied strain ε_{app} is defined as

$$\varepsilon_{app} = \frac{2\delta_0}{L} \qquad \textbf{Equation S3}$$

Therefore a straight ribbon of length L should have a uniform strain of ε_{app} if the end effects are neglected. Attributing to symmetry and antisymmetry, a unit cell can ultimately be represented by a quarter cell with a fixed boundary condition at the axis of symmetry and a displacement of $\delta_0/2$ at the end. The reaction force is denoted as F_0. The end of the unit cell is assumed to be free to rotate, because the unit cell is extracted from a long, periodic serpentine ribbon whose boundary conditions do not influence the unit cell.

Effective stiffness, k_{eff}, is defined as the ratio between the reaction force F_0 and the effective displacement, $2\delta_0$. The actual structure of the fabricated serpentine unit cell is constructed with three layers as follows: (1) a half unit cell with an arc radius R_1, mechanically connected to a half unit cell with an arc radius R_3; (2) a unit cell with an arc radius R_2; and (3) a half unit cell with an arc radius R_3 mechanically connected to a half unit cell with an arc radius R_1. The three layers are laminated together to form a complex serpentine unit cell. The reaction forces of unit cells with different arc radii R_1, R_2, and R_3 are denoted as F_1, F_2, and F_3, respectively. Two serpentine unit cells mechanically connected in series have the same reaction force regardless of the geometric parameters.

$$F_1 = F_3 \qquad \textbf{Equation S4}$$

The relationship between the reaction force F_0 of the entire complex multilayer unit cell and the reaction force of each unit cell is described as follows.

$$F_0 = F_2 + 2F_1 = F_2 + 2F_3 \qquad \textbf{Equation S5}$$

And the corresponding tensile displacements at each end have a relationship as follows.

$$2\delta_0 = \delta_{0,1} + \delta_{0,3} = 2\delta_{0,2} \qquad \textbf{Equation S6}$$

The effective stiffnesses of these three unit cells with different arc radii R_1, R_2, and R_3 are denoted as $k_{eff,1}$, $k_{eff,2}$, and $k_{eff,3}$, respectively. Based on the definition of effective stiffness:

$$k_{eff,1} = \frac{F_1}{2\delta_{0,1}}$$ **Equation S7.1**

$$k_{eff,2} = \frac{F_2}{2\delta_{0,2}}$$ **Equation S7.2**

$$k_{eff,3} = \frac{F_3}{2\delta_{0,3}}$$ **Equation S7.3**

Rearranging these expressions gives:

$$F_1 = 2k_{eff,1}\delta_{0,1}$$

$$F_2 = 2k_{eff,2}\delta_{0,2}$$

$$F_3 = 2k_{eff,3}\delta_{0,3}$$

Substituting the forces into **Eq. S5**, we obtain:

$$F_0 = F_2 + 2F_1 = 2k_{eff,2}\delta_{0,2} + 2\left(2k_{eff,1}\delta_{0,1}\right) = 2k_{eff,2}\delta_{0,2} + 4k_{eff,1}\delta_{0,1}$$ **Equation S8**

Since

$$\delta_0 = \delta_{0,2}$$

We obtain:

$$F_0 = 2k_{eff,2}\delta_0 + 4k_{eff,1}\delta_{0,1}$$ **Equation S9**

From

$$F_1 = F_3$$

Substituting stiffness definitions (**Eq. S7**) gives:

$$k_{eff,1}\delta_{0,1} = k_{eff,3}\delta_{0,3}$$ **Equation S10**

Using displacement compatibility, from **Eq. S6**

$$\delta_{0,1} + \delta_{0,3} = 2\delta_0$$

We obtain:

$$\delta_{0,3} = 2\delta_0 - \delta_{0,1}$$

Substituting into **Eq. S10** yields,

$$k_{eff,1}\delta_{0,1} = k_{eff,3}\left(2\delta_0 - \delta_{0,1}\right) = 2k_{eff,3}\delta_0 - k_{eff,3}\delta_{0,1}$$

Thus,

$$\delta_{0,1} = \frac{2k_{eff,3}}{k_{eff,1}+k_{eff,3}}\delta_0$$ **Equation S11**

Recall **Eq. S9**:

$$F_0 = 2k_{eff,2}\delta_0 + 4k_{eff,1}\delta_{0,1}$$

Substituting **Eq. S11** into **Eq. S9** yields:

$$F_0 = 2k_{eff,2}\delta_0 + 4k_{eff,1}\left(\frac{2k_{eff,3}}{k_{eff,1} + k_{eff,3}}\delta_0\right) = 2k_{eff,2}\delta_0 + \frac{8k_{eff,1}k_{eff,3}}{k_{eff,1} + k_{eff,3}}\delta_0$$

The effective stiffness is therefore:

$$k_{eff} = \frac{F_0}{2\delta_0} = \frac{1}{2\delta_0}\left(2k_{eff,2}\delta_0 + \frac{8k_{eff,1}k_{eff,3}}{k_{eff,1}+k_{eff,3}}\delta_0\right) = k_{eff,2} + \frac{4k_{eff,1}k_{eff,3}}{k_{eff,1}+k_{eff,3}}$$ **Equation S12**

Section S9. Modification of a Commercial Melt-Based Material Extrusion (*m*-MEX) 3D Printer for Printing Flexible TPU Filament

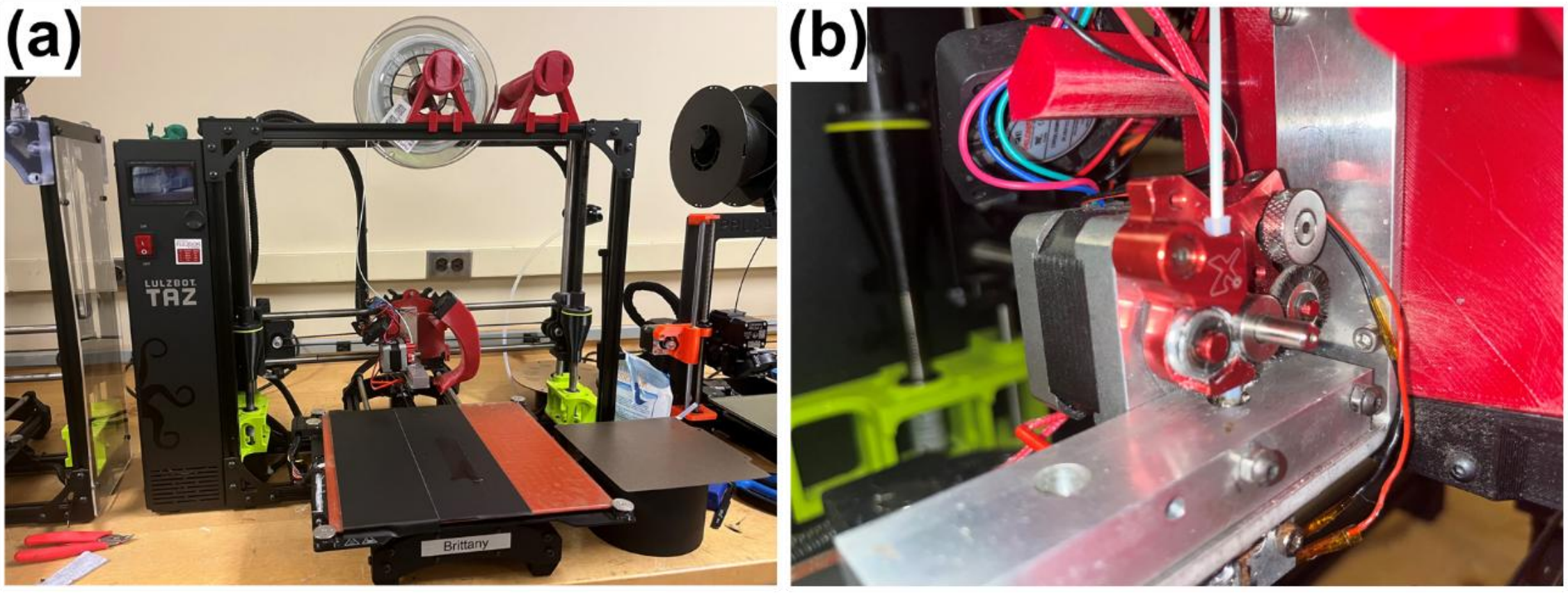

FIGURE S11. Modified melt-based material extrusion (*m*-MEX) 3D printing platform. (a) Commercial LulzBot TAZ 6 3D printer modified for *m*-MEX printing using an X60 ultra-flexible TPU filament. (b) Flexion™ extruder optimized for flexible filaments and installed on the LulzBot TAZ 6 printer to enable stable extrusion of soft TPU materials.

Section S10. Supplementary Movie Caption

MOVIE S1. Soft gripper demonstration.

References

1. Su, Q., Zou, Q., Li, Y., Chen, Y., Teng, S.-Y., Kelleher, J.T., Nith, R., Cheng, P., Li, N., Liu, W., Dai, S., Liu, Y., Mazursky, A., Xu, J., Jin, L., Lopes, P., and Wang, S. (2021) A stretchable and strain-unperturbed pressure sensor for motion interference–free tactile monitoring on skins. *Sci. Adv.*, **7** (48), eabi4563.
2. Xia, X., Xiang, Z., Gao, Z., Hu, S., Zhang, W., Long, R., Du, Y., Liu, Y., Wu, Y., Li, W., Shang, J., and Li, R. (2024) Structural Design and DLP 3D Printing Preparation of High Strain Stable Flexible Pressure Sensors. *Advanced Science*, **11** (37), 2304409.
3. Yuan, Y., Xu, H., Zheng, W., Liu, M., Li, S., Yan, J., Wang, D., Liu, K., Zhang, H., Chen, G., Wang, W., Wu, G., Xue, C., Cheng, H., and Gao, L. (2024) Bending and Stretching-Insensitive, Crosstalk-Free, Flexible Pressure Sensor Arrays for Human-Machine Interactions. *Adv Materials Technologies*, **9** (5), 2301615.
4. Yang, C., Su, H., Zhang, S., Ji, H., Qi, Z., Wang, Y., Cheng, E., Zhao, L., and Hu, N. (2025) High Strain-Insensitive Performance Stretchable Pressure Sensor Based on the Laser-Engraved Graphene with a Serpentine Nested Structure. *ACS Appl. Nano Mater.*, **8** (2), 1129–1136.
5. Ha, K.-H., Li, Z., Kim, S., Huh, H., Wang, Z., Shi, H., Block, C., Bhattacharya, S., and Lu, N. (2024) Stretchable hybrid response pressure sensors. *Matter*, **7** (5), 1895–1908.
6. Li, C., Zhang, J., Yu, H., Zhao, F., Xu, Z., Wei, X., Wang, H., Chen, X., Ye, Z.-G., Zhang, X., and Liu, M. (2026) Deformation-adaptive pressure sensors based on multi-level discrete sensing arrays for morphing electronics and human–machine interaction. *npj Flex Electron*, **10** (1), 21.
7. Dong, S., Zhu, X., Guo, Y., Zhang, P., Ma, G., Li, W., and Zhang, S. (2025) A Stretch-Insensitive Pressure Sensor Based on Liquid Metal Composite with a Hierarchical Conductive Network. *ACS Appl. Mater. Interfaces*, **17** (27), 39480–39489.
8. Guo, Y., Dong, S., Lu, H., Gong, L., Zhang, Q., Zhou, X., Zhang, S., Du, H., and Li, W. (2025) Anisotropic hierarchical conductive network for stretch-insensitive tactile sensor with ultra-sensitivity and a broad detection range. *Cell Reports Physical Science*, **6** (12), 102963.
